# Age-dependent impact of two exercise training regimens on genomic and metabolic remodeling in skeletal muscle and liver of male mice

**DOI:** 10.1038/s41514-022-00089-8

**Published:** 2022-06-27

**Authors:** Michel Bernier, Ignacio Navas Enamorado, Mari Carmen Gómez-Cabrera, Miguel Calvo-Rubio, Jose Antonio González-Reyes, Nathan L. Price, Ana Belén Cortés-Rodríguez, Juan Carlos Rodríguez-Aguilera, Sandra Rodríguez-López, Sarah J. Mitchell, Kelsey N. Murt, Krystle Kalafut, Katrina M. Williams, Christopher W. Ward, Joseph P. Stains, Gloria Brea-Calvo, Jose M. Villalba, Sonia Cortassa, Miguel A. Aon, Rafael de Cabo

**Affiliations:** 1 Translational Gerontology Branch, National Institute on Aging, NIH, Baltimore, MD 21224 USA; 2 Freshage Research Group, Department of Physiology, Faculty of Medicine, University of Valencia, and CIBERFES, Fundación Investigación Hospital Clínico Universitario/INCLIVA, Valencia, Spain; 3 Departamento de Biología Celular, Fisiología e Inmunología, Campus de Excelencia Internacional Agroalimentario, ceiA3, Universidad de Córdoba, Campus de Rabanales, Edificio Severo Ochoa, 3ª planta, 14014 Córdoba, Spain; 4 Servicio de Fisiopatología Celular y Bioenergética, Universidad Pablo de Olavide, Sevilla, 41013 Spain; 5 Department of Orthopaedics, University of Maryland School of Medicine, Baltimore, MD 21201 USA; 6 Centro Andaluz de Biología del Desarrollo and CIBERER, Instituto de Salud Carlos III, Universidad Pablo de Olavide - CSIC – JA, Sevilla, 41013 Spain; 7 Laboratory of Cardiovascular Science, National Institute on Aging, NIH, Baltimore, MD 21224 USA; 8 Present Address: Translational Medicine Section, Akouos, Inc., 645 Summer St, Boston, MA 02210 USA

**Keywords:** Ageing, Metabolism

## Abstract

Skeletal muscle adapts to different exercise training modalities with age; however, the impact of both variables at the systemic and tissue levels is not fully understood. Here, adult and old C57BL/6 male mice were assigned to one of three groups: sedentary, daily high-intensity intermittent training (HIIT), or moderate intensity continuous training (MICT) for 4 weeks, compatible with the older group’s exercise capacity. Improvements in body composition, fasting blood glucose, and muscle strength were mostly observed in the MICT old group, while effects of HIIT training in adult and old animals was less clear. Skeletal muscle exhibited structural and functional adaptations to exercise training, as revealed by electron microscopy, OXPHOS assays, respirometry, and muscle protein biomarkers. Transcriptomics analysis of gastrocnemius muscle combined with liver and serum metabolomics unveiled an age-dependent metabolic remodeling in response to exercise training. These results support a tailored exercise prescription approach aimed at improving health and ameliorating age-associated loss of muscle strength and function in the elderly.

## Introduction

Aging is characterized by a progressive deterioration in organismal homeostasis that underlies many chronic diseases in the elderly. Age-related health deficits predict increased risk for negative health outcomes, including morbidity and mortality, in both mice and humans^1^. An age-related decline of muscle mass and strength, known as sarcopenia, contributes to markers of physical frailty, such as slow gait, fatigue, and muscle weakness^2–4^. Thus, it is important to investigate interventions with the potential to prevent skeletal muscle decline and increase healthy lifespan in aging populations.

The age-associated loss of muscle mass is associated with remodeling of skeletal muscle structure and metabolism in both humans and rodent models^5–7^. Several molecular changes, including defects in mitochondrial biogenesis^8,9^, bioenergetics^10,11^, dynamics^12^, and respiratory capacity^13,14^ contribute to the deterioration of muscle structure and function during aging. The induction of protein synthesis and mammalian target of rapamycin complex 1 (mTORC1) signaling in response to nutrients, insulin, and growth factors is reduced in skeletal muscle of older adults^15–18^. Skeletal muscle insulin resistance also contributes to impaired systemic glucose homeostasis and age-related metabolic dysfunction^8^. Skeletal muscle lipid accumulation has also been associated with decreased strength in older individuals^19^, and changes in glucose and lipid metabolism have been observed in the skeletal muscle of aged mice^20^. Successful interventions will likely target these diverse molecular features of aging skeletal muscle.

Exercise is a well-established intervention that can prevent many of the health defects and chronic diseases associated with aging^21^. The benefits of exercise training can be traced to multiple organ systems, but exercise is a particularly attractive intervention for age-related metabolic dysfunction in skeletal muscle^22^. Investigation into the mechanisms underlying the beneficial effect of exercise training on skeletal muscle has revealed stimulation of muscle protein synthesis irrespective of age^18,23,24^ and reversal of skeletal muscle insulin resistance, resulting in an increased induction of anabolic mTORC1 signaling^25^. Aerobic exercise also improves markers of mitochondrial function and increases respiratory supercomplex assembly in human skeletal muscle^9,26–28^. Frequent and regular physical exercise also induces beneficial changes in skeletal muscle bioenergetics in terms of ATP production, NAD^+^ homeostasis, and pyrimidine nucleotide biosynthesis^29^. Because exercise has the potential to improve skeletal muscle quality and strength and combat muscle dysfunction, a better understanding of the mechanisms and potential therapeutic responses to various exercise modalities is of broad basic and clinical interest.

Training intensity and duration determine the predominant fuel source utilized by skeletal muscle. Carbohydrate-derived substrates provide most of the energy during high-intensity exercise, whereas plasma fatty acids supply most of the energy during lower intensity aerobic exercise^30^. Furthermore, early stages of exercise are characterized by oxidation of intramuscular glycogen and triglyceride stores, while uptake and oxidation of plasma glucose and fatty acids from liver and adipose tissues, respectively, are increased progressively during prolonged bouts^30^. Metabolic waste products from skeletal muscle, namely lactate and glycerol, can subsequently serve as gluconeogenic precursors to support hepatic glucose production^30^. Thus, skeletal muscle metabolism is supported by continuous crosstalk with the liver and adipose tissues. Yet, how skeletal muscle and systemic adaptation to different training regimens are affected by age is largely unknown.

Recent studies suggest the amount of time spent exercising declines with age^31^, which stems from the perceived lack of time for physical activity^32^. Alternating periods of intense exercise (defined as >70–75% VO_2_ max) and active recovery, also known as high-intensity interval training (HIIT), significantly improve cardiorespiratory fitness and elicit the same physiological response as traditional moderate-intensity continuous training (MICT) with less time commitment^33^. Here, this study was aimed at comparing the adaptive responses of adult (5 months) and old (24 months) male C57BL/6 J mice to two types of daily exercise training (HIIT vs. MICT) to evaluate the potential of each regimen to combat age-related muscle weakness and metabolic dysfunction. Results from phenotypic, biochemical, and unbiased transcriptomic analyses from mouse gastrocnemius muscle indicate that 4-week MICT training elicited substantial benefits in old mice along with hepatic metabolic remodeling needed to provide energetic substrates to muscle. These results support the use of continuous moderate aerobic exercise training to improve muscle function and metabolic health in the elderly.

## Results

### Impact of different exercise regimes on physiological and physical activity of adult and old mice

**Fig. 1:**
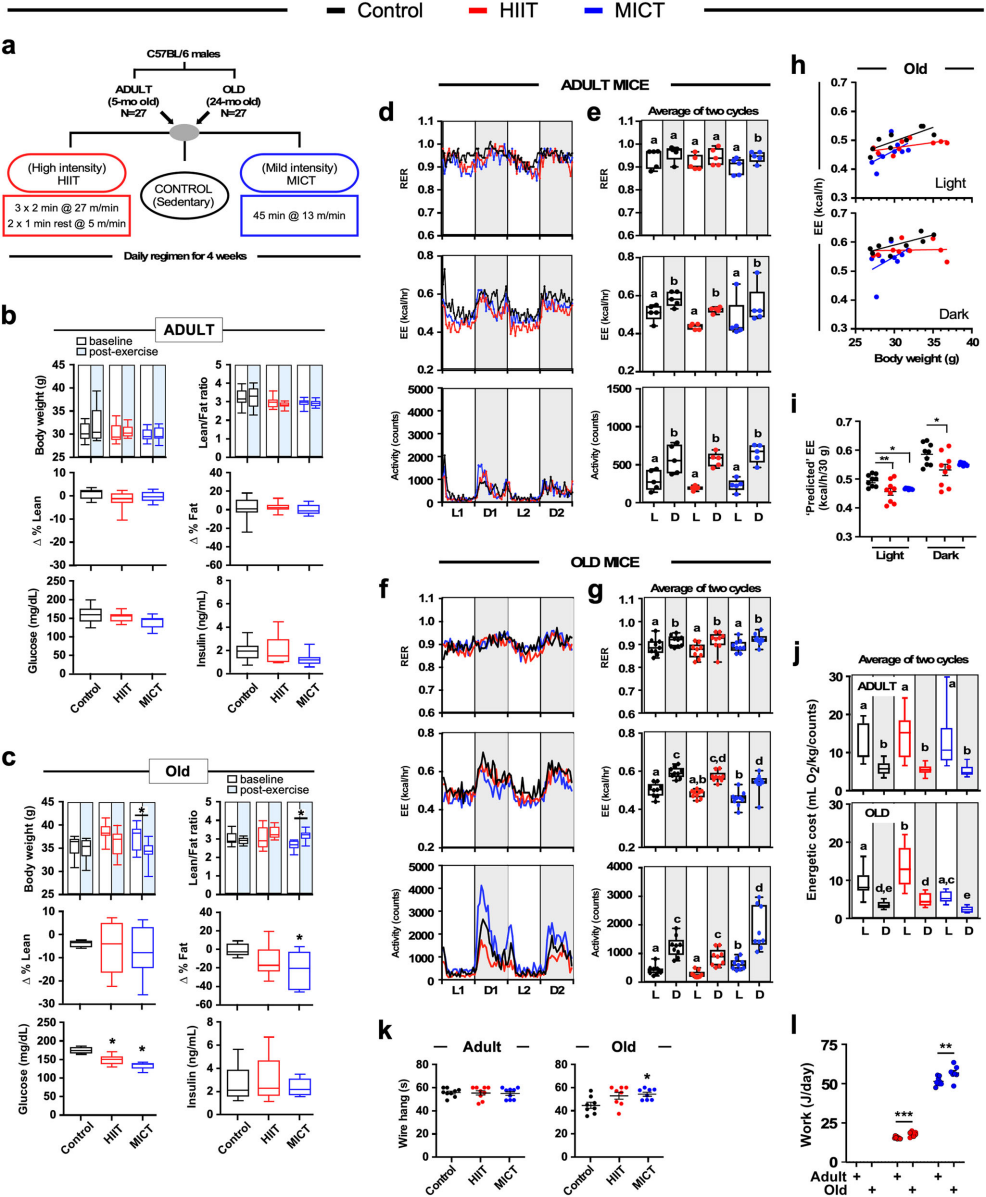
Impact of HIIT and MICT exercise training on whole-body physiology and in vivo metabolism in adult and old mice. **a** Schematic of the experimental protocol. **b**, **c** *Upper panels*, body weight and lean-to-fat ratio at baseline (white boxes) and at the conclusion of the 4-week training period (blue boxes) in adult (**b**) and old (**c**) mice. *Middle panels*, changes in lean and fat percentages from baseline as determined by NMR. *Bottom panels*, at the conclusion of the training period, mice were fasted for 6 h for the measure of blood glucose and insulin levels, *n* = 9 mice per group. **d**–**g** Sedentary and exercised mice were placed in metabolic cages to measure their respiratory exchange ratio (RER) [e.g., ratio of the volume of carbon dioxide generated (VCO_2_) over the volume of oxygen consumed (VO_2_)], energy expenditure (EE) and voluntary ambulatory activity over 60 h, as detailed in the Method section. **d**, **f** Hourly trajectories over 48 h in adult (**d**) and old (**f**) mice; L light phase, D dark phase, **e**, **g** Values associated with the two light (L1 + L2) and dark (D1 + D2) cycles were averaged and shown as box and whisker plots with individual adult (**e**, *n* = 5 per group) and old (**g**, *n* = 9 per group) mice. One-way ANOVA with Tukey’s post-hoc test was carried out to test for significance. Two variables with different lowercase letters have a statistically significant relationship (with *p* ≤ 0.05): light vs. dark phase; young vs. old; sedentary vs. exercised groups. **h** Relationship between EE and body mass in old sedentary and exercised mice during the light and dark cycles (*n* = 9 per group). Data have been analyzed using ANCOVA with EE as dependent variable, exercise as fixed variable, and body mass as covariate. The regression line equations can be found in Supplementary Table 2. **i** Scatter plots are depicting mean ± SEM. Significance levels represent the projected exercise effect on EE from the ANCOVA analysis at a given body mass of 30 g. **j** The energetic cost of locomotion was determined from VO_2_ per unit of body weight and locomotor activity in counts. Data from two light (L1 + L2) and dark (D1 + D2) cycles were collected and shown as box and whisker plots, *n* = 5 mice per group. **k** Time to fall from a wire hang was assessed at the conclusion of the training period, *n* = 9 mice per group. **l** Daily work during the 4-week training period was expressed as Joules/day. Data have been analyzed considering body weight (kg) × total vertical distance traveled (defined as treadmill speed (m/min) × % grade × exercise time (min)). *n* = 5 mice per group. The 5-point box and whisker plots depict minimum, lower quartile (Q1), median (Q2), upper quartile (Q3), and maximum values. No outliers were excluded from the presented data. One-way ANOVA with Tukey’s post-hoc test was carried out to test for significance. *p ≤ 0.05; **p ≤ 0.01; ***p ≤ 0.001.

Two different exercise training protocols were carried out daily toward the end of the light cycle (between 4:00–5:00 PM) in adult (5 months) and old (24 months) C57BL/6 J male mice (*n* = 9 per experimental group), as schematized in Fig. 1a. The treadmill training consisted of daily sessions during four consecutive weeks with HIIT comprising three short runs (2 min each) at 27 m/min with a one-min of active recovery (5 m/min) between bouts, and MICT involving a single 45-min run at 13 m/min (see Methods for further details).

The food consumption of the sedentary controls and exercised mice did not change during the training period (in g/day: adult, 3.0–3.5; old, 2.5–3.0). The workflow chart depicted in Supplementary Fig. 1a describes the testing procedure for the adult and old cohorts of mice. Compared to baseline, adult HIIT- and MICT-trained mice did not exhibit changes in body weight, percentage of lean mass and body fat, or lean-fat ratio (Fig. 1b), whereas old MICT-trained animals lost weight and decreased their % body fat, resulting in higher lean-fat ratios (Fig. 1c). A clear age difference in morphometric measures between adult and old MICT mice was recorded in this cross-sectional study (Supplementary Fig. 1b). A large variability in body composition metrics (e.g., % lean and body fat) was observed in old exercised vs. sedentary control mice (Fig. 1c vs. b). Old, but not adult mice, exhibited reduced levels of the 6-h fasting blood glucose (FBG) 24 h after the last training session (Fig. 1b, c). Blood lactate (Supplementary Fig. 1c) and serum insulin (Fig. 1b, c) levels as well as HOMA-IR values (Supplementary Fig. 1d) did not change in response to exercise training, regardless of age.

Because aging impacts the energetic cost of movement and substrate utilization^34^, we quantitated in vivo metabolic function and free-living activity via indirect calorimetry experiments in metabolic cages. Here, the simultaneous measurements of oxygen consumed (VO_2_) and CO_2_ exhaled (VCO_2_) per h, respiratory exchange ratio (RER), energy expenditure (EE, expressed as kcal/h), and cage activity counts were quantified. Hourly trajectories of these metrics were captured for 60 h, discarding the first 12-h acclimation period and retaining two 24-h cycles (L1-D1-L2-D2) (Fig. 1d, f). Overall, mice from the sedentary and exercised groups displayed distinctive diurnal patterns of RER, EE, and cage activity, consistent with their ability to adapt to changes in metabolic demand. The measurements recorded across the two light and dark cycles were averaged (Fig. 1e, g), and showed old sedentary mice exhibiting lower RER than their adult counterparts (light cycle, 0.889 ± 0.036 vs. 0.937 ± 0.042, *p* = 0.046; dark cycle, 0.914 ± 0.025 vs. 0.961 ± 0.035, *p* = 0.012; mean ± SD with unpaired two-tailed Student’s *t*-test), indicating an increased utilization of fats, consistent with age-based differences in substrate metabolism^35^. The RER values were significantly lower during the light vs. dark cycles in old mice (Fig. 1g, *upper panel*), regardless of the type of exercise, while in the adult mice RER cycling was observed only in response to MICT (Fig. 1e, *upper panel*). Compared to their age-matched sedentary controls, adult mice on HIIT and MICT regimens had significantly smaller areas under the RER curve (RER-AUC) than old mice (Supplementary Table 1). The EE-AUC was also significantly lower with exercise training in adult mice vs. sedentary controls, whereas old mice exhibited much smaller EE-AUC with MICT vs. HIIT regimen (Supplementary Table 1). Since body weight is an important contributor to EE and some differences in body weight were observed in our experimental groups we assessed whether the relationship between body weight and EE was statistically different between the experimental groups by using regression-based analysis-of-covariance (ANCOVA)^36,37^. The regression line equations of each group of mice are presented in Supplementary Table 2 (see Supplementary Fig. 1f and Fig. 1h for graphical illustration). Moreover, because the deviation of each data point from the regression line is preserved under ANCOVA^38^, it enables visualization of group differences at a given body weight. The results showed that the predicted EE based on a fixed body weight, arbitrarily set at 30 g, was reduced in old mice in response to both HIIT and MICT (Fig. 1i), but not in adult mice (Supplementary Fig. 1g). Therefore, while both adult and old animals show reduced overall EE, only old animals show significant changes when the impact of body weight is accounted for.

Although the cage activity-AUC was comparable between exercised adult mice and their sedentary controls, old mice that performed MICT were significantly more active than old HIIT and sedentary groups (Supplementary Table 1). These observations led us to assess the energetic cost of locomotion in each group of mice placed in metabolic cages by considering the volume of O_2_ consumed, body weight of the animal, and cage activity. The energetic cost of locomotion was minimally impacted regardless of age or exercise regimen in the averaged light and dark cycles, although a significant reduction was observed when directly comparing MICT to HIIT in old mice (Fig. 1j). These results indicate that the selective decrease in body weight in old MCT mice is likely the primary factor responsible for the reduction in EE in these animals.

We then assessed the effect of the 4-week training period on neuromuscular performance. Contrary to adult mice, old MICT-trained mice showed an increase in hanging time from the hanging wire test (Fig. 1k), which could be accounted for by their lower body mass. These results together with the lack of significant change in forepaw grip strength, without and with body weight normalization (Supplementary Fig. 1e), suggest no improvement in functional performance in response to these exercise training protocols.

The two exercise regimen protocols used in this study differ in intensity (speed) of running, but also in duration. To determine whether some of the adaptations noted may be linked more to the amount of work done rather than intensity, we calculated treadmill work done, considering treadmill speed and incline, the body weight of the animal, and the duration of the run (see Methods for more details). The results indicate a far greater amount of work was done with MICT vs. HIIT, with old mice working significantly harder than their adult counterparts (Fig. 1l), which stems from the fact that the older mice were heavier (in g, 34.2 ± 2.92 (old) vs. 30.1 ± 1.43 (adult), *p* = 1.10e-05 by Student’s *t*-test).

Taken together, the results indicate that a 4-week MICT regimen in old mice reduces body mass and improves glucose regulation associated with lower energy expenditure and increased voluntary locomotor activity and neuromuscular performance with a slight effect on RER. We surmise that in old mice, MICT confers broad benefits vs. sedentary mice and those performing HIIT, whereas the two exercise paradigms did not elicit detectable adaptation in adult animals.

### Structural and functional impact of exercise training on mitochondria in skeletal muscle

We sought to gain insight into whether exercise training elicited metabolic and functional adaptations in the morphology and function of mitochondria in the skeletal muscle.

Transmission electron microscopy (TEM) of fiber bundles was used to profile cross-sections of red and white parts of the gastrocnemius by examining relevant ultrastructural features: sarcolemma, myofibrils, sarcoplasmic reticulum, and mitochondria in both adult (Supplementary Fig. 2a–f, m–r) and old (Supplementary Fig. 2g–l, s–x) mice in response to exercise training. As expected, mitochondria were more abundant in red fibers and differentially located just beneath the sarcolemma–subsarcolemmal mitochondria or SSM- or in the intermyofibrillar region—intermyofibrillar mitochondria or IMF. Representative micrographs from SSM and IMF regions are depicted in Supplementary Fig. 3a and b, respectively. Images were taken at 25,000X magnification and enlarged figures depicted in Supplementary Fig. 2a–x are found in Source data file 1.

**Fig. 2:**
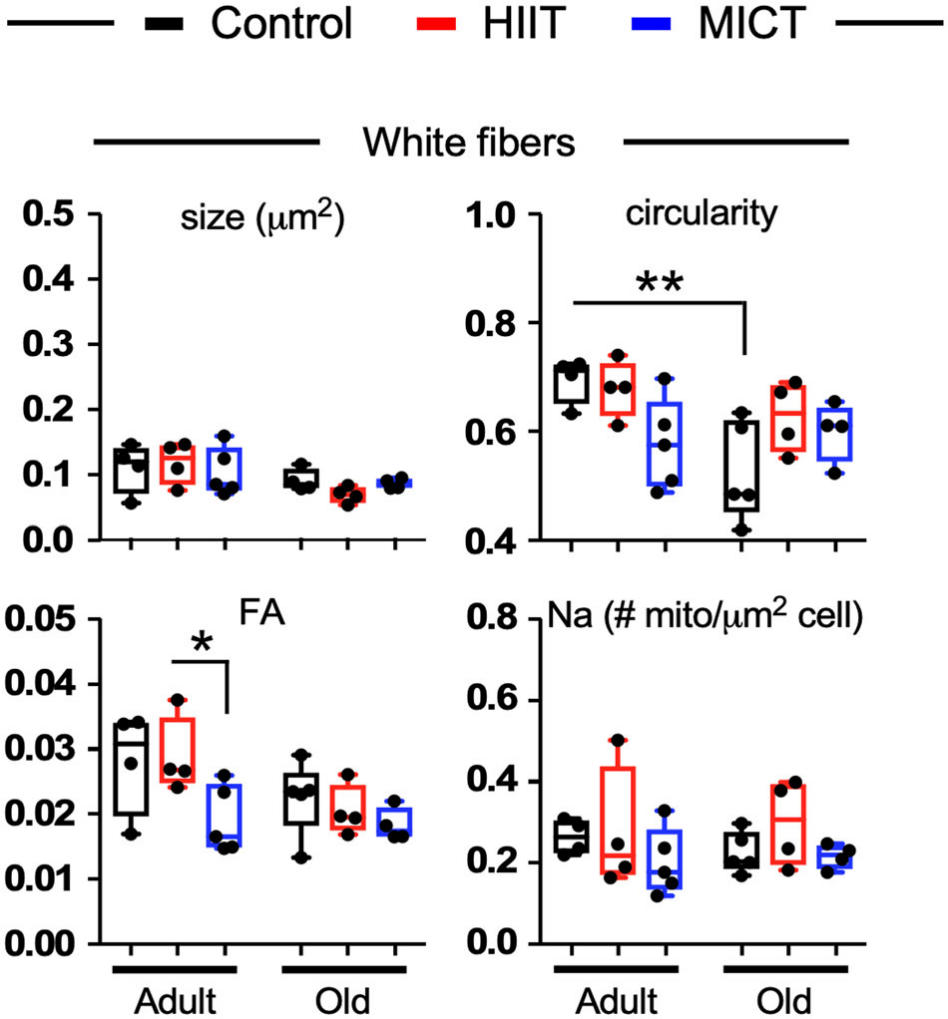
Morphometric and stereological assessment of mitochondria in white muscle fibers of adult and old mice. Mitochondrial size and circularity of mitochondria (upper panels) from adult and old sedentary mice or mice subjected to a 4-week daily exercise regimen, HIIT or MICT. Fractional area (FA) of mitochondria and numerical density profiles are depicted in the lower panels. *n* = 4–5 animals per condition. Data are represented as box and whisker plots, depicting minimum, lower quartile (Q1), median (Q2), upper quartile (Q3), and maximum values. Significance between groups was determined using Two-way ANOVA with Sidak’s post-hoc tests. *p ≤ 0.05; **p ≤ 0.01.

Altered mitochondrial size and shape is linked to impairment in mitochondrial function^39,40^. In white fibers, a significant decrease in circularity was detected in old vs. adult sedentary mice without alteration in mitochondrial size (Fig. 2, *upper right and left panels*, and Supplementary Fig. 2), consistent with an improvement in mitochondrial function^39,40^. The number of mitochondria per μm^2^ of cell area (Na) was not impacted by exercise training, regardless of age (Fig. 2, *bottom right panel*) whereas the fractional area (Fa) of the cell occupied by mitochondria was 33.7 ± 5.4% lower in adult MICT *vs*. HIIT mice (Fig. 2, *bottom left panel*). In red fibers, TEM parameters of IMF (Supplementary Fig. 4, *upper panels*) and SSM (Supplementary Fig. 4, *middle panels*) mitochondria and their Fa (Supplementary Fig. 4, *bottom left panel*) remained unchanged in response to age or training. However, a 61.3 ± 14.1% higher mitochondrial density (Na) with HIIT was found when comparing old vs. adult mice (Supplementary Fig. 4, *bottom right panel*). Together, the data show lack of a significant influence of exercise training on mitochondrial morphology and abundance suggesting that these exercise regimens may be more directly affecting mitochondrial function.

**Fig. 3:**
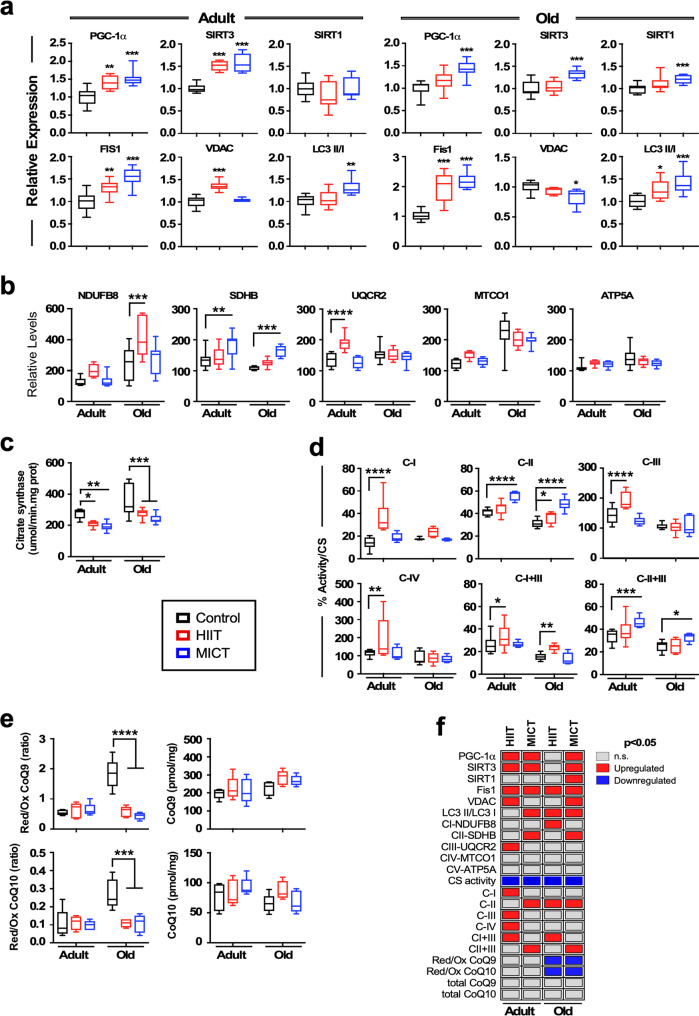
Impact of exercise training on mitochondrial dynamics in skeletal muscle. **a** Muscle lysates (10 μg per lane) were immunoblotted for key markers of mitochondrial dynamics (PGC-1α, SIRT3, Fis1, VDAC), SIRT1, and autophagosomal activity (LC3-II/LC3-I ratio). Quantitation of protein levels after data normalization with Ponceau S staining of nitrocellulose membranes. *n* = 8 mice per group. Original gels and stained membranes are shown in Supplementary Fig. 5a. **b** Same lysates were immunoblotted for key components of mitochondrial OXPHOS (complex I, NDUFB8; complex II, SDHB; complex III, UQCR2; complex IV, MTCO1; complex V, ATP5A). *n* = 8 mice per group. Original gels are shown in Supplementary Fig. 5b. **c** Citrate synthase activity in muscle tissue homogenates, *n* = 5 mice per group. **d** Determination of electron transport chain activities (C-I, C-II, C-III, C-IV, C-I + III, and C-II + III) measured in total homogenates preparations and normalized by citrate synthase, *n* = 5 mice per group. **e** *Left panels*, Ratio of the different forms of ubiquinol (reduced CoQ_9_H_2_ and CoQ_10_H_2_) and ubiquinone (oxidized CoQ_9_ and CoQ_10_); *right panels*, CoQ was extracted from quadricep muscle tissues and resolved by HPLC, *n* = 5 mice per group. ***p ≤ 0.001. **f** Graphical representation of the influence of HIIT and MICT vs. age-matched sedentary controls regarding various mitochondrial bioenergetic parameters in adult and old mice. The data shown in **a**–**e** are expressed as box-and whisker plots, depicting minimum, lower quartile (Q1), median (Q2), upper quartile (Q3), and maximum values. No outliers were excluded from the presented data. Comparisons between control and experimental groups (HIIT or MICT) were performed using one-way ANOVA followed by Dunnett’s post-hoc tests. *p ≤ 0.05; **p ≤ 0.01; ***p ≤ 0.001.

The impact of exercise training on mitochondrial protein content was further assessed by measuring the expression of select proteins implicated in the status of posttranslational acetylation (SIRT1 and SIRT3), mitochondrial fission (Fis1), autophagy/mitophagy (Fis1, LC3), biogenesis (PGC-1α), and energetics (VDAC1) (Fig. 3a and Supplementary Fig. 5a). In muscle extracts from exercised adult (in response to HIIT and MICT) and old MICT mice, there was significantly higher abundance of the transcriptional coactivator of mitochondrial biogenesis PGC-1α. In adult mice, under both exercise regimens SIRT3 was increased, whereas higher levels of SIRT3 and SIRT1 were observed in old MICT mice. The levels of Fis1, a mitochondrial fission and upstream factor for mitophagy^41^, were significantly higher with exercise training in adult and old mice, and the voltage-dependent anion channel 1 (VDAC1), an outer membrane transporter of adenine nucleotides and regulator of cell survival^42^, was elevated in adult HIIT mice while being lower in the old MICT group. The microtubule-associated protein 1 A/1B-light chain 3 (LC3)-II, a commonly used marker of autophagosomal activity^43^, displayed a significantly greater LC3-II/LC3-I ratio in adult MICT-trained mice and exercised old mice compared to their respective sedentary controls (Fig. 3a). Together these data suggest accumulation of proteins responsible for mitochondrial biogenesis, mitophagy, energetics, and autophagosomal activity upon exercise training.

Next, we assessed the impact of exercise training on distinct aspects of mitochondrial function. Moderate-intensity training elicits the assembly of mitochondrial respiratory chain complexes in human skeletal muscle resulting in higher respiratory flux^28^. To investigate this, we determined the abundance of mitochondrial OXPHOS complexes in gastrocnemius extracts immunoblotted with antibodies specific for protein components of each mitochondrial complex including: NADH dehydrogenase (ubiquinone) 1 beta subcomplex subunit 8 (NDUFB8 or complex I), succinate dehydrogenase [ubiquinone] iron-sulfur subunit (SDHB or complex II), cytochrome b-c1 complex subunit 2 (UQCR2 or complex III), cytochrome c oxidase subunit 1 (MTCO1 or complex IV), and ATP synthase (ATP5A or complex V). HIIT-trained adult mice exhibited higher expression of UQCR2 protein, while NDUFB8 protein was more abundant in old HIIT-trained mice, with SDHB being elevated under MICT regardless of age (Fig. 3b and Supplementary Fig. 5b). Exercise training did not affect the levels of complexes IV and V proteins in adult or old muscles.

Next, we measured the in vitro activity of respiratory complexes (Supplementary Table 3) normalized to the citrate synthase activity, which was significantly decreased in skeletal muscle of adult and old exercised mice (Fig. 3c). In adult mice, the relative activity of complexes I, III, IV, and I + III was significantly higher with HIIT, whereas MICT increased complex II and II + III activities (Fig. 3d). Old, HIIT-trained mice had greater complex II and I + III activities, while MICT training elicited higher complex II and II + III activities (Fig. 3d). The ratio of reduced-to-oxidized forms of coenzyme Q (CoQH_2_/CoQ) is considered a probe of respiratory chain efficiency, i.e., the lower the ratio the higher the efficiency^44,45^. Herein, the CoQH_2_/CoQ ratio for coenzyme Q_9_ or Q_10_ was higher in muscle of sedentary old *vs*. adult mice, and exercise training effectively diminished both ratios in old, but not adult animals, where it exerted no effect (Fig. 3e). The total pools of CoQ_9_ and CoQ_10_ were not significantly modified by age or exercise training.

### Impact of exercise training on the skeletal muscle transcriptome

The benefits of exercise training on the overall energetic behavior of mice, the specific remodeling of the mitochondrial respiratory chain, and relevant aspects in mitochondrial metabolism, e.g., acetylation, mitophagy, biogenesis, and fission, led us to hypothesize that adaptation to a particular type of exercise will be different in old vs. adult mice through enrichment of sets of genes implicated in select biological processes. To this end, we performed gene expression microarray analysis of gastrocnemius samples at the conclusion of the 4-week training period.

**Fig. 4:**
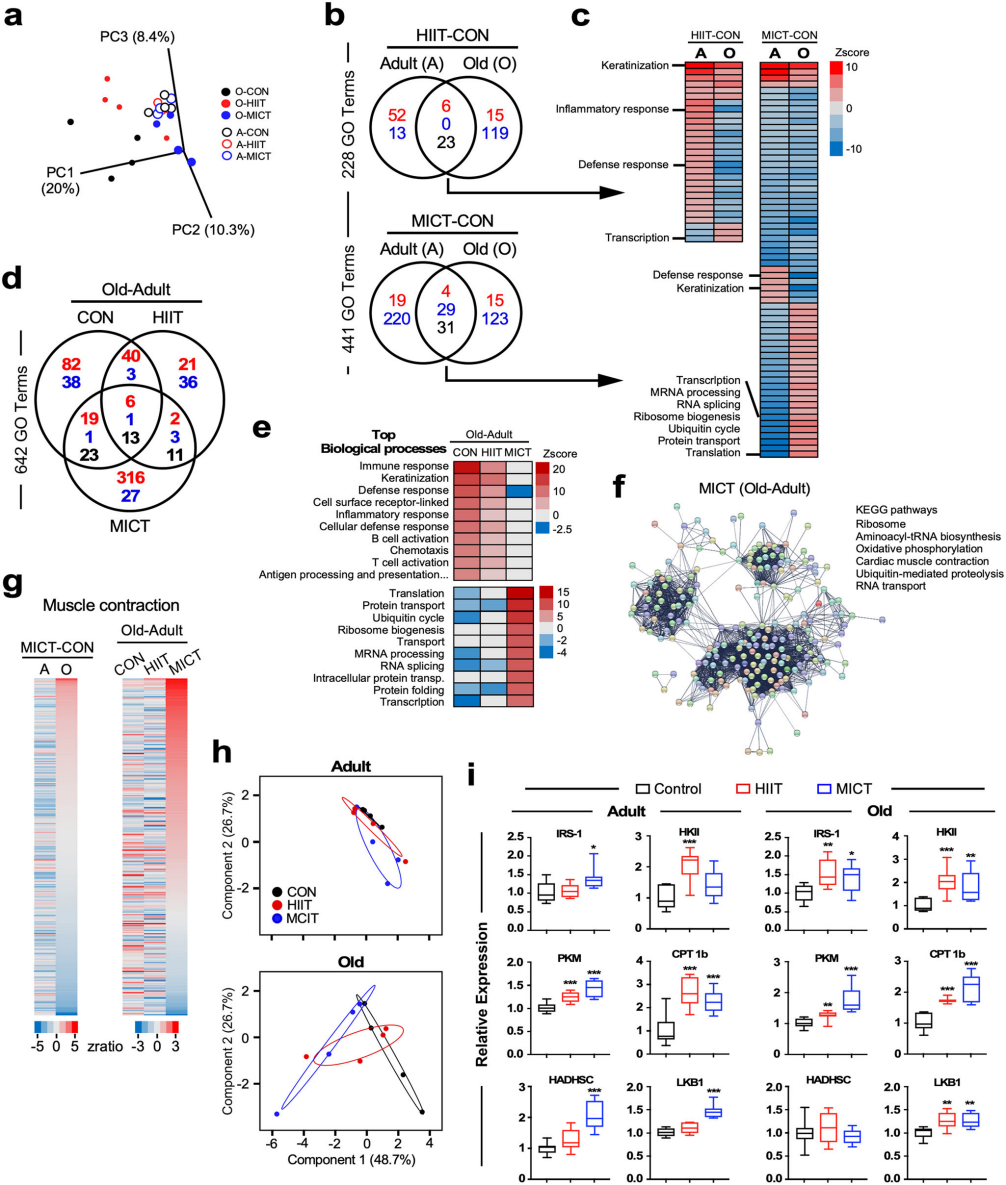
Transcriptomics analysis of skeletal muscle from adult and old mice subjected to HIIT or MICT regimen. **a** Principal component analysis (PCA) from microarray experiments. Global gene expression profile revealed the impact of exercise (HIIT and MICT) in adult (A, open symbols) and old (O, filled symbols) mice vs. sedentary controls (CON). A-CON, *n* = 4; A-HIIT, *n* = 3; A-MICT, *n* = 3; O-CON, *n* = 4; O-HIIT, *n* = 4; O-MICT, *n* = 4. **b** Venn diagrams depicting the number of unique and shared GO Terms (biological processes) between adult (A) and old (O) mice in the (HIIT-CON, upper panel) and (MICT-CON, lower panel) pairwise comparisons; red font, upregulated; blue font, downregulated; black font, reciprocal regulation. **c** Heatmaps of the shared GO Terms derived from (**b**) in the (HIIT-CON, *n* = 29) (left panel) and (MICT-CON, *n* = 64) (right panel) pairwise comparisons. A comprehensive list of the shared biological processes is provided in Supplementary Table 4. Significant GO Term enrichment was defined by both *Z*-score > 1.5 in either direction and −log (*P*) > 1.30. **d** Venn diagram depicting the number of unique and shared GO Terms (biological processes) present in the (Old-Adult) pairwise comparison from sedentary (CON), HIIT and MICT groups of mice; red font, upregulated; blue font, downregulated; black font, reciprocal regulation. **e** Heatmap of top biological processes upregulated in sedentary (CON, upper panel) or MICT (lower panel) mice in the ‘Old-Adult’ pairwise comparison. A comprehensive list of the enriched biological processes is provided in Supplementary Table 5. Significant GO Term enrichment was defined by both *Z*-score > 1.5 in either direction and −log (*P*) > 1.30. **f** STRING database analysis depicting potential interactions among differentially expressed genes (color-filled symbols) present in the Old vs. Adult MICT mice and encompassing the GO terms ‘electron transport’, ‘ubiquitin cycle’, and ‘translation’. The various colors are for visualization only. A list of the top KEGG pathways and associated false-discovery rates (FDR) is provided. **g** Heatmaps depicting the impact of age in MICT mice (left panel) and that of exercise in the (Old-Adult) pairwise comparison (right panel) vis-à-vis enrichment of the GO term, muscle contraction (GO:0006936). **h** Validation of the microarray data using real-time PCR. **i** Densitometric quantification of IRS-1, HKII, PKM, CPT1b, HADHSC, and LKB1 proteins after data normalization with Ponceau S staining of nitrocellulose membranes. Immunoblot images from EDL homogenates are depicted in Supplementary Fig. 6. Data are shown as box and whisker plots, depicting minimum, lower quartile (Q1), median (Q2), upper quartile (Q3), and maximum values. and. No outliers were excluded from the presented data. Comparisons between control and experimental groups (HIIT or MICT) were performed using Students *t*-test. *p ≤ 0.05; **p ≤ 0.01; ***p ≤ 0.001 vs. sedentary controls. *n* = 8 mice per group.

Principal component analysis (PCA) revealed that the muscle transcriptome of sedentary (CON) old mice was distinct to that of adult mice (filled vs. open black symbols) (Fig. 4a). Exercise training had a minimal impact on skeletal muscle gene expression in the adult; conversely, some form of adaptation to either type of exercise was observed in the muscle transcriptome of old mice, with the old MICT muscle being somewhat closer to the adult (Fig. 4a). We performed a pairwise comparison ‘HIIT-CON’ and ‘MICT-CON’ to identify the exercise-dependent gene sets significantly impacted as a function of age (Fig. 4b). The shared groups of Gene Ontology (GO) terms represent biological processes impacted by HIIT or MICT vs. sedentary controls in both adult and old animals. A significant proportion of these GO terms moved in the opposite direction (23/29 for HIIT and 31/64 for MICT) between adult and old mice, as visualized in Fig. 4c (for complete list, see Supplementary Table 4), indicating the clear impact of age and type of exercise on ‘core’ processes in skeletal muscle transcriptome. MICT was the most effective at eliciting changes in gene set enrichment. Noteworthily, pathways related to keratinization, defense response, and transcription followed the same signature in HIIT-CON and MICT-CON pairwise comparison, whereas processes associated with proteostasis, RNA splicing, and translation were singularly upregulated in old mice post-MICT training.

We then performed a pairwise comparison ‘Old-Adult’ to identify the age-dependent gene sets significantly impacted by the exercise regimens (Fig. 4d). Once again, a higher number of changes in gene set enrichment were observed within the MICT group. Pathways related to ‘inflammatory/defense/ immune’ response were the predominant mark in old vs. adult sedentary controls, whereas biological processes related to translation, RNA splicing/spliceosome, and proteostasis were significantly upregulated in response to MICT (Fig. 4e and Supplementary Table 5a). In contrast, the HIIT intervention somewhat corrected the profile of the old sedentary muscle without the functional benefits of MICT (Supplementary Table 5b). Heatmaps of differentially expressed genes from select biological processes were generated and showed a clear beneficial impact of MICT *vs*. CON and HIIT in the old vs. adult mice (Supplementary Fig. 6a).

Results thus far show the emergence of age-associated pathways centered on inflammation/immune response in sedentary old vs. adult controls. We show that while HIIT and MICT exercise regimes suppressed this response in old mice, improvement in translation/proteostasis and mitochondrial energetics underscored the functional benefit of the MICT intervention. To effectively visualize the interconnections between these pathways, we used the differentially expressed genes from ‘Electron transport’, ‘Ubiquitin cycle’ and ‘Translation’ as input for the STRING functional network association program (https://string-db.org/) and applied a high strength reliability cut-off of 0.7 (Fig. 4f). This network demonstrated a very high enrichment probability (PPI, *p* < 1.0e-16) and the interactome was further analyzed for emerging pathways using the KEGG module database. The three major clusters describe a clear pattern of predominant pathways comprising proteostasis- (aminoacyl tRNA biosynthesis, ribosome, ubiquitin-mediated proteolysis) and energy-related (OXPHOS, muscle contraction) processes in response to MICT training in old skeletal muscle. The set of genes associated with muscle contraction (GO:0006936) offered a unique signature, with MICT in the ‘Old-Adult’ comparison, returning a *Z*-score = 5.1611, *p*-value = 8.75e-07, and fdr −1.03e-05. (Fig. 4g): Among the top 10 genes significantly impacted were Trim63, Aldoa, Tcap, Tnnc2, Tnni2, and Myom2 (upregulated), and Chrna3, Ghsr, Cxcr4, F2r (downregulated).

A set of eight genes were used for validation of the microarray using qPCR (Supplementary Fig. 6b and Supplementary Table 6). PCA of *Z*-score normalized qPCR data revealed extensive overlap between the three adult groups whereas old mice on MICT were separated to some extent from HIIT and sedentary controls (Fig. 4h). The exercise-regulated transcriptional regulation of inflammation in skeletal muscle led us to perform multiplex quantification of cytokines in muscle extracts (Supplementary Table 7). A significant age-dependent increase in KC/GRO (CXCL1) was observed in old MICT-trained muscle, while expression of IL-10 was higher in old mice following HIIT protocol vs. sedentary controls and intramuscular IL-6 levels were elevated in adult MICT males vs. controls (Supplementary Fig. 6c, Supplementary Table 8). Immunoblotting for fibronectin, collagen III, and α-smooth muscle actin (SMA) showed no significant change in their expression among the experimental groups (Supplementary Fig. 6d, e), indicating the lack of post-exercise fibrotic alterations to the muscle.

Overall, these findings support the idea that extensive genetic reprogramming underlies the salutary impact of MICT in the skeletal muscle of old mice. To further validate these results, we assessed the expression of key players in muscle growth signal (IRS-1), glucose metabolism (hexokinase II (HKII), pyruvate kinase muscle isozyme (PKM)), mitochondrial β-oxidation (carnitine palmitoyl transferase I (CPT1b), short-chain l-3-hydroxyacyl-CoA dehydrogenase HADHSC), and maintenance of energy homeostasis (liver kinase B1 (LKB1)) by immunoblotting using gastrocnemius extracts prepared from mice 36–48 h after completing the training protocols (Fig. 4i and Supplementary Fig. 6f for immuno-stained membranes). We found that old mice were just as responsive as their adult counterparts to the benefits of the two exercise modalities. Adult MICT-trained mice had significantly higher levels of IRS-1 than CON and HIIT groups, while IRS-1 levels were upregulated in the old-exercised groups. The HKII protein was markedly accumulated in muscle from adult HIIT-trained mice and in exercised old muscle vs. age-matched sedentary CON. A significant increase in PKM and CPT1b protein levels was found in both adult and old muscles after exercise training, whereas significant accumulation of HADHSC was only observed in adult MICT muscle. Lastly, LKB1 levels were upregulated with exercise training.

Together, the transcriptome data and the expression of associated molecular markers indicate an extensive reprogramming of gene expression in the response of skeletal muscle to different exercise regimens as a function of age. This reprogramming involves processes directly related to mitochondrial metabolism, such as electron transport, and the proteostasis network, both contributing to the sustainability of the energetic response of skeletal muscle to exercise training across the lifespan.

### The impact of exercise training on liver and serum metabolomics during aging

To further investigate and validate the response of skeletal muscle to exercise training at the organism level, we determined the hepatic and serum metabolomes as a function of age and exercise regimes. We aimed at investigating whether, beyond muscle, exercise training elicited metabolic remodeling in the liver, as a main supplier of substrates, via serum, to mainly catabolic organs such as heart and brain. The increased fuel demands of the muscle during exercise training requires the participation of hepatic metabolism regulated in part by substrate supply through integration of carbohydrate, fat, and amino acid metabolic pathways^46,47^.

**Fig. 5:**
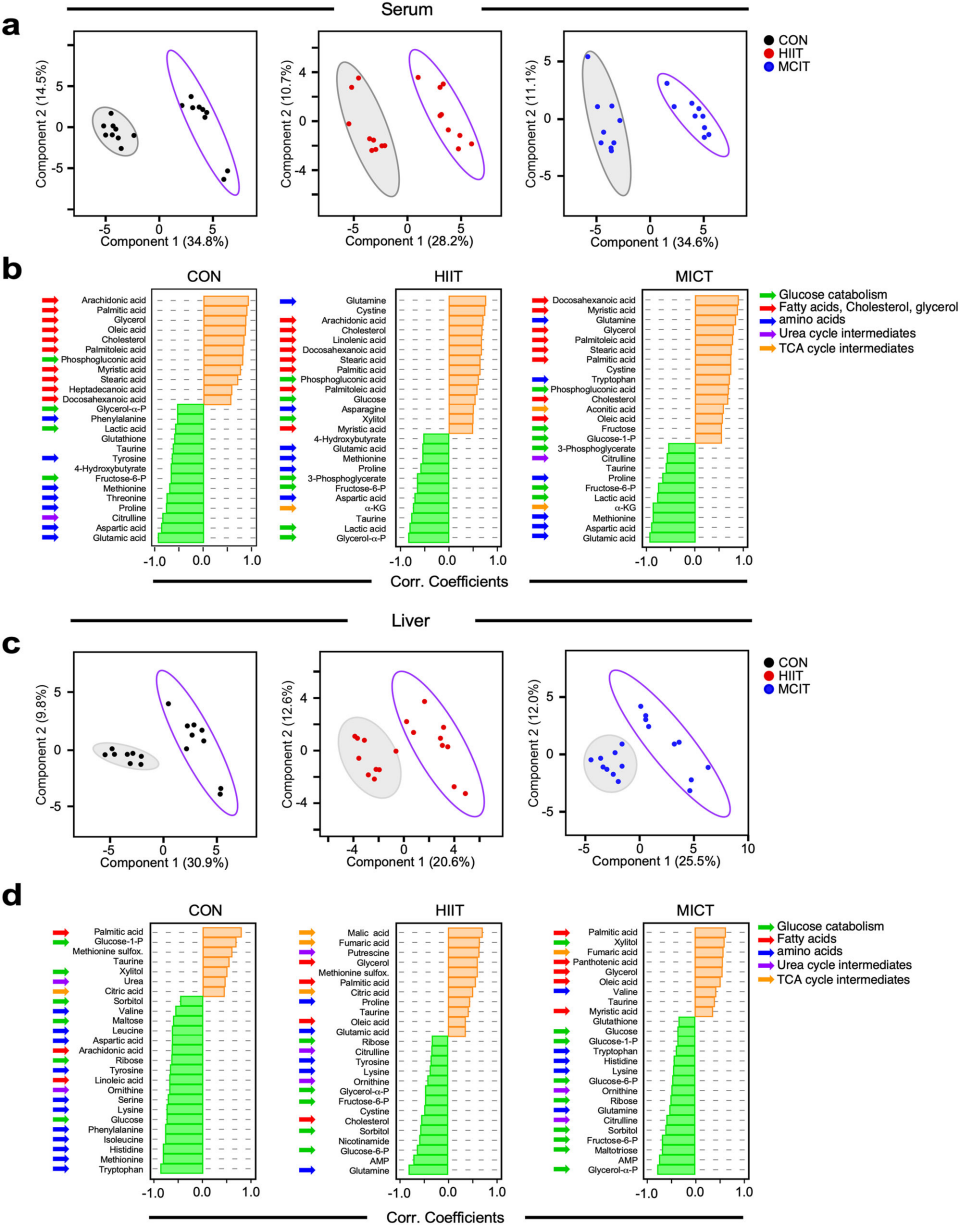
Age-dependence analysis of untargeted metabolomics performed in serum and liver from sedentary and exercised mice. **a** Serum and **c** liver metabolite profiles from sedentary controls (left panels), HIIT (middle panels), and MICT (right panels) mice were analyzed by Partial Least Square Determinant Analysis (PLS-DA). The ellipses correspond to 95% confidence intervals for a normal distribution. Each principal component is labeled with the corresponding percent values. Gray-filled ellipses, adult; non-filled purple ellipses, old. **b**, **d** Correlation coefficients of the top 25 metabolites that correlated positively (orange bars) and negatively (green bars) in function of age for serum (**b**) and liver (**d**).

Untargeted mass spectrometry metabolomics was conducted on serum and liver from the same groups of mice (Fig. 1a). Profiles comprising 171 metabolites that were detected and identified in both serum and liver were analyzed, first, for age-dependent metabolic differences within each exercise regime (e.g., sedentary (CON), HIIT, and MICT) in adult *vs*. old mice by partial least square discriminant analysis (PLS-DA), a supervised clustering method. Overall, we found a clear separation between adult and old mice within each exercise regime group in both serum and liver (Fig. 5a, c).

To identify the main metabolites responsible for age-dependent metabolic differences as a function of the exercise regimen, we analyzed the age dependence (adult < old) of changes in metabolite patterns by quantifying the Pearson correlation coefficient, with positive (enrichment, orange bars) and negative (depletion, green bars) correlation values for each type of exercise training (CON, HIIT, MICT) (Fig. 5b, d), with the pattern being strikingly different in CON *vs*. the exercised groups. For instance, the liver from old mice in the CON group presents a prominent depletion of metabolites from AA metabolism (Fig. 5d, *left panel*), whereas that pattern was more mixed in the HIIT and MICT groups, which displayed more nuanced differences in the enrichment/depletion patterns. The liver enrichment pattern of metabolites in HIIT animals comprised intermediates from the tricarboxylic acid (TCA) cycle (malic, fumaric, citric) and a mixed depletion pattern that includes AAs (tyrosine, lysine, glutamine) or AA-related (citrulline, ornithine) and glucose (ribose, fructose 6 P, glucose 6 P, glycerol α-P) metabolism (Fig. 5d, *middle panel*). The MICT regime (mild and more sustained in time than in HIIT) presented a pattern of metabolite enrichment in liver from old animals characterized by lipids (palmitic, oleic, myristic) or lipid-related (pantothenic, glycerol) metabolism, and a pronounced depletion pattern of glucose (glucose, glucose 1 P, glucose 6 P, ribose, sorbitol, fructose 6 P, maltotriose, glycerol α-P) metabolism. The pattern enrichment of metabolites from serum was significant for lipids in the three experimental groups (Fig. 5b), whereas the depletion pattern of metabolites was more AA-related in CON (Fig. 5b, *left panel*), and mixed (AAs, glucose) in HIIT and MICT (Fig. 5b, *middle* and *right panels*, respectively). Heatmaps of the metabolites that account for the separation between CON, HIIT, and MICT metabolic profiles from the PLS-DA analysis are depicted in Supplementary Fig. 7a, c, while those showing average values of the metabolites enriched in serum and liver as a function of exercise training are found in Supplementary Fig. 7b, d, respectively.

**Fig. 6:**
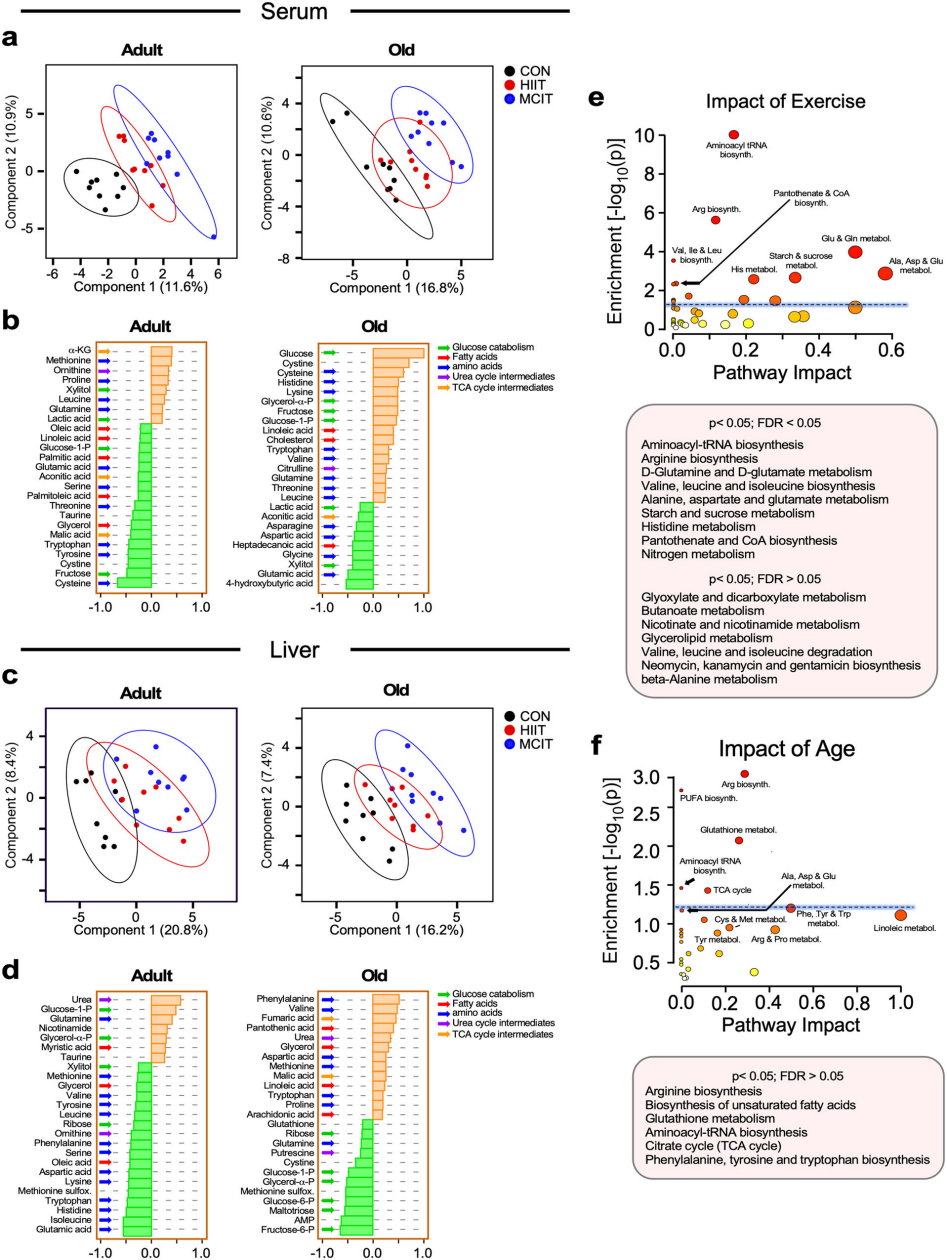
Exercise training impact analysis of untargeted metabolomics performed in serum and liver from adult and old mice. **a** Serum and **c** liver metabolite profiles from adult (left panel) and old (right panel) mice were analyzed by Partial Least Square Determinant Analysis (PLS-DA). The ellipses correspond to 95% confidence intervals for a normal distribution. Each principal component is labeled with the corresponding percent values. Black symbols, Sedentary controls; red symbols, HIIT; blue symbols, MICT. **b**, **d** Correlation coefficients of the top 25 metabolites that correlated positively (orange bars) and negatively (green bars) as a function of exercise for serum (**b**) and liver (**d**). The impact of exercise (**e**) and age (**f**) vis-à-vis pathway enrichment and their network topology from the liver metabolome is depicted. *y*-axis, enrichment significance; *x*-axis, pathway impact for network topology. Blue line highlights pathways significantly impacted as defined by enrichment significance *p* ≤ 0.05 [−log (*p*) > 1.3] and fdr ≤ 0.05.

Next, to identify the metabolites associated with the exercise-dependent (CON, HIIT, MICT) metabolic differences within each age group (adult and old), we performed PLS-DA followed by correlation pattern analysis. Overall, CON and the MICT exercised group were better separated than the HIIT group, which exhibited an extensive overlap with the other two groups, although the overlap was higher in liver (Fig. 6c) than in serum (Fig. 6a). Unlike in adult liver, where there was still considerable overlap between MICT and sedentary control groups, in the old liver, this exercise regimen did result in a clear separation between those same groups (MICT and sedentary) (Fig. 6c). Comparatively, these results agree with the idea that age appears as a stronger discriminant than exercise regimen among experimental groups (compare Fig. 6a, c with Fig. 5a, c).

The identity of the main metabolites responsible for exercise-dependent metabolic differences in each age group was assessed through the changes in metabolite patterns with exercise training (CON < HIIT < MICT) as quantified by Pearson correlation coefficients (Fig. 6b, d). The liver of adult mice displayed a mixed pattern of positive correlations represented by a few metabolites from glucose or the glucose-fatty acid cycle (glucose 1 P, glycerol α-P), and amino acids (AAs) (urea, glutamine) metabolism, and a more prominent pattern of metabolites’ negative correlations, i.e., depletion from pathways related to AA metabolism (methionine, branched chain—valine, isoleucine, leucine–tyrosine, serine, phenylalanine, aspartic, lysine, tryptophan, histidine, glutamic) (Fig. 6d). This metabolic pattern was consistent with serum depletion of AAs (glutamic, serine, threonine, tryptophan, tyrosine, cysteine, cystine) in adult mice (Fig. 6b), suggesting that the liver acted as one of the main consumer organs. In stark contrast, the liver from old, exercised mice (compared to their adult counterparts) exhibited an inverse metabolic pattern characterized by a prominent enrichment of AAs or AA-related metabolites (phenylalanine, valine, aspartic acid, methionine, proline, urea, fumaric) along with lipids or lipid-related (linoleic, arachidonic, pantothenic, glycerol) metabolism. Importantly, this pattern was concomitant with liver depletion of metabolites from glucose metabolism (ribose, glucose 1 P, glycerol α-P, glucose 6 P, fructose 6 P, maltotriose). The serum metabolome from old, exercised mice mirrored their liver metabolic pattern to a certain extent showing both enrichment (e.g., cysteine, cystine, histidine, lysine, tryptophan, valine) as well as depletion of AAs (asparagine, aspartic, glycine, glutamic) or AA-related (citrulline) metabolism (Fig. 6b).

To further dissect the effect of age from exercise training on liver metabolism, we performed two-way ANOVA (Supplementary Fig. 8 and 9). We sought to find a select group of metabolites which variance was significantly affected only by age *vs*. exercise training alone or in interaction with age. Each select group of metabolites was subjected to pathway analysis using MetaboAnalyst 5.0 that employs enrichment and network topology metrics (e.g., relative betweenness centrality). We found that exercise training, either alone or interacting with age, affected (*p* < 0.05) several pathways prominently related with AAs, including protein translation (aminoacyl tRNA synthesis), and lipid (pantothenic, CoA, glycerolipid, butyric) metabolism (Fig. 6e). Specifically, pathway analysis revealed that the prevalent effect of exercise training on AA metabolism was to elicit biosynthesis of non-essential AAs (e.g., arginine, alanine, aspartate, glutamate, glutamine) and utilization of essential AAs (histidine, BCAAs -valine, isoleucine, leucine-), as well as AA degradation (BCAAs). The convergence of AA catabolism on mitochondria feeds anabolism through hepatic gluconeogenesis via glucogenic AAs (e.g., glutamate, histidine, arginine, alanine) as well as the generation of ketone bodies from ketogenic AAs (e.g., BCAAs).

The select group of hepatic metabolites affected by age rendered a varied group of significant pathways that included lipid (synthesis of unsaturated fatty acids, ω6-polyunsaturated linoleic), AAs (arginine, alanine, aspartate, glutamate, phenylalanine, tyrosine, tryptophan), protein translation (aminoacyl tRNA synthesis), and redox-related (glutathione, cysteine-methionine) metabolism (Fig. 6f).

The ensemble of metabolomics data in liver and serum is consistent with the idea that exercise training triggers a specific metabolic remodeling in liver, characterized by pathways of AA and lipid metabolism which is distinct to the one generated in specific response to age alone (Fig. 6e vs. f). In liver, exercise training promoted biosynthesis of non-essential AAs that along with essential AAs, likely supplied by diet or protein degradation, both contributed to protein translation and specific catabolism of BCAAs. Overall, this is consistent with the amphibolic nature of this organ, along with gluconeogenesis and ketone body generation at the level of mitochondria^48^. The metabolite patterns of liver from old- compared to adult-exercised mice were in stark contrast, consisting of prominent enrichment of AAs, or AA-related, along with lipid, or lipid-related, metabolism, and liver depletion of metabolites from glucose metabolism, suggesting a shift in substrate selection, i.e., from glucose to lipid/AAs (Fig. 6d and Supplementary Fig. 7f). The serum metabolome from old exercised mice mirrored to a certain extent the liver metabolic pattern of old mice, showing both enrichment as well as depletion of AAs and AA-related metabolism (Fig. 6b and Supplementary Fig. 7e). Importantly, the pattern of metabolite enrichment in liver from old MICT mice, characterized by lipid and lipid-related metabolism, is consistent with a more expected engagement of mitochondrial oxidative phosphorylation (i.e., because of prolonged MICT) and the respiratory chain remodeling exhibited by mitochondria from skeletal muscle (Fig. 3).

### Global hierarchical clustering

**Fig. 7:**
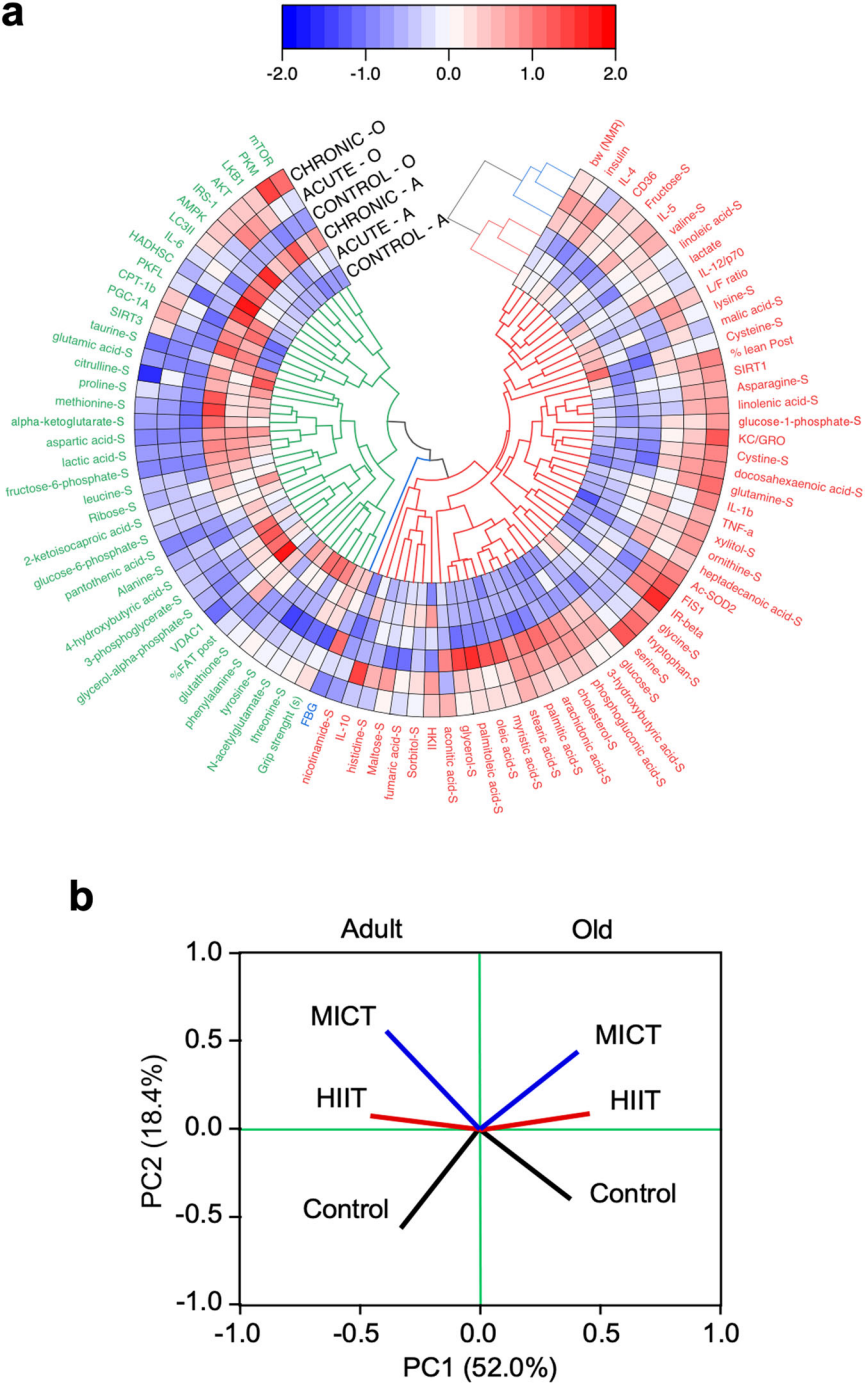
Integrated interpretation of various data sets that include phenotypical, physiological, biochemical, and metabolomics markers. **a** Polar heatmaps depicting hierarchical clustering and **b** principal component analysis (PCA) of Z-score normalized physiological, biochemical and serum metabolomics data based on values from the six experimental groups using uncentered similarity metrics and average linkage. The pseudocolor scale of **a** is a warming scale corresponding to the normalized values (blue and red, denoting low and increasing values, respectively) with respect to the age average of each column’s values ranging between -2 and +2 according to the bar on the top. Molecular biomarkers of inflammation, mitochondrial function, and turnover along with nutrient signaling pathways and serum metabolome reflect the impact of age and exercise regimen as the major and second principal components separating the experimental groups, respectively.

To understand the systemic impact of age and exercise on healthspan, we generated polar heatmaps depicting hierarchical clustering of the Z-normalized physiological, metabolic, energetic, and mitochondrial biochemical-functional data from skeletal muscle, as well as serum metabolomics data (Fig. 7a). A good separation between adult and old mice, and between sedentary controls vs. exercise-trained animals was achieved with this approach, which further illuminates the relative beneficial advantage of MICT in old vs. adult mice. More specifically, old groups showed higher dependence on lipids and redox metabolism along with differential abundance of proinflammatory cytokines as a function of exercise compared to their adult counterparts, the latter exhibiting more amino acid and glucose metabolism converging to mitochondria, including mitochondrial turnover. Principal Component Analysis of the same input data showed ‘age’ to be the strongest principal component (PC1 = 52.0%), while PC2 (18.4%) described changes associated with training regimen (Fig. 7b), supporting the notion of a beneficial outcome to exercise training, especially MICT, across age.

## Discussion

The present cross-sectional study in adult (5 months) and old (24 months) male mice addresses functional differences associated with a 4-week exercise training period of varying intensity (HIIT vs. MICT) while monitoring underlying mechanisms at multiple levels, including physiological, physical, metabolic, energetic, and ‘molecular-biochemical-omics’ levels. The ensemble of data shows that exercise training, particularly MICT, elicits salutary effects in old *vs*. adult mice, as the following main findings indicate: *(i)* enhanced fitness (higher cage activity, wire hanging) and physiological performance (body weight reduction, higher lean/fat ratio, lower fasting blood glucose, energy expenditure, and energetic cost of locomotion); *(ii)* a consistent pattern of advantageous effects bestowed by the MICT regime to mitochondrial biochemical and functional metrics in skeletal muscle of old *vs*. adult mice; *(iii)* specific genomic remodeling triggered by exercise training, as revealed by biomarkers-validated transcriptomics in skeletal muscle leading to differential gene expression linked to muscle growth and function involving translation, proteostasis, spliceosome activity, inflammation/immunity, and energetics (e.g., mitochondrial respiration); *(iv)* remodeling of the liver metabolome dependent on the modality of exercise performed, with a distinct pattern of metabolites from old *vs*. adult mice in response to exercise training. Together, the ensemble of data is consistent with exercise training eliciting specific genomic (skeletal muscle) and metabolic (liver) adaptations directed to life-sustaining processes of energy generation and balance (mitochondrial metabolism—respiration, TCA cycle, lipid, and AA degradation) and regeneration (amino acids and protein translation/synthesis, proteostasis, mRNA splicing). The systemic impact of both age and exercise training on healthspan is further revealed by a hierarchically integrated overview of changes in key physiological and molecular processes involved in mitochondrial function, nutrient signaling, metabolism, and inflammation (Fig. 7).

Most research on HIIT has been done in young and middle-aged subjects, and as such, the effects in senior populations are less known. Most common primary outcomes in the HIIT studies describe changes in cardiorespiratory fitness (such as VO_2_ peak) as well as feasibility and safety of the protocols^49^. The transcriptomic and, more specifically, metabolomic changes induced by HIIT in old individuals are largely unknown, with few exceptions^50^ where levels of several genes related to mitochondria, insulin signaling, and muscle growth were downregulated with age in skeletal muscle biopsies. These data demonstrate a varied response of gene transcripts based on exercise modalities between young and older adults, with the greatest increase corresponding to HIIT in older individuals.

Relatively few studies have provided insight into the specific effects of exercise training and age on whole-body energetics and voluntary activity in mice. Indirect calorimetry data revealed that exercised adults utilize more lipids than carbohydrates (CHO) as substrates compared to sedentary controls, a finding consistent with the fact that longer, continuous exercise training (e.g., MICT) requires more energy supply. However, old mice in response to MICT had a higher mixed substrate use (CHO & lipids) and showed a reciprocal pattern of low EE with high locomotor activity. A significant reduction in energetic cost of locomotion was observed in old mice after 4 weeks of MICT *vs*. HIIT, but not in adult mice. The greater treadmill work done in response to MICT *vs*. HIIT training is consistent with some of the physiological/metabolic adaptations noted in this study through an underlying remodeling of gene expression and metabolism in contracting skeletal muscle and substrate supply from liver.

Although changes in morphology were modest, mitochondria from skeletal muscle exhibited significant functional improvements, especially from MICT-trained 24-month-old male mice, as revealed by up-modulation of biomarkers of biogenesis (PGC-1α), energetics (VDAC1), SIRT3 deacetylase, fission (FIS1) and autophagosomal LC3-II/LC3-I, an indicator of increased mitochondrial recycling. Moreover, signatures of elevated mitochondrial function consistent with enhanced respiratory flux from complex II concomitant with enhanced CoQ reoxidation at complex III correlated with enhanced levels of the complex II component SDHB and activity of complex II and CII + III along with weaker CoQ Red/Ox ratio. As a caveat, since complex II and II + III activities were increased with exercise training regardless of age, but no such increases were always observed for complex I, III, and IV-dependent activity, rules out the potential artifact introduced by the normalization of respiratory complex activity with respect to citrate synthase activity. Additionally, the expected down-modulation of the Red/Ox ratio of CoQ resulting from higher respiratory flux through complex II was found in the MICT regimen.

Proteomics from healthy adults revealed that better oxidative capacity in skeletal muscle associates with an enrichment of major protein clusters implicated in mitochondrial energetic functions, mitochondrial translation, and the mRNA alternative splicing machinery, independent of age, sex, and physical activity^51^. Temporal coherence between the transcriptome and metabolome underlies the benefits of proper exercise timing in skeletal muscle from sedentary and exercised adult mice, revealing a higher enrichment of lipid metabolism in early rest phase after training in the sedentary group and a switch to glycolysis with exercise training^52^. Here, we found that skeletal muscle from old MICT mice exhibits a gene enrichment pattern consisting of pathways related to “RNA splicing”, “protein modification process”, “protein folding and transport”, and “translation” *vs*. sedentary and HIIT groups, accompanied by a sharp reduction in pro-inflammatory/immune processes. These findings lead us to propose that exercise regimens, especially MICT, can provoke a metabolic rearrangement to cope with the effects of aging on the energetic function of skeletal muscle.

Daily physical activity is associated with suppressed immune activity in adults; however, there are contradictory views on the subject^53–55^. Our study shows a significant enrichment of genes related to inflammation and immunity-related pathways in muscle of old sedentary mice and their suppression with MICT. The cellular mechanisms responsible for the age-associated mitochondrial dysfunction of skeletal muscle in mice correlate significantly with higher levels of mitochondrial oxidative damage^56,57^. Indeed, the association of aging with a multi-organ functional decline in mammals is accompanied by alterations in oxidative stress, inflammation, and nutrient signaling pathways, ultimately affecting the quality and health in aged muscle^58^.

Liver metabolomics displayed a select group of metabolites responding to exercise training and age, both alone and in combination, uncovering a prevalent effect of exercise training on hepatic AA metabolism — specifically, biosynthesis of non-essential AAs in addition to BCAA degradation—while age-dependent metabolites presented a distinct pattern of pathways, which was more diverse compared to those responding to exercise training. A great deal of changes in serum metabolome, as compared to the liver, suggested the participation of other organs in the provision or uptake of metabolites from the circulation.

### Study limitations and future prospects

An important limitation of the present study is that it was carried only in male C57BL/6 J mice. Whether the benefits of exercise training can be reproduced in male and female mice of different genetic backgrounds is unclear at this point and warrants further investigation. It is known that skeletal muscle physiology, muscle perfusion, and voluntary activity have a sex-dependent component^59^, and sex differences were seen in hepatic gene expression between sedentary and exercised mice^60^. In this regard, it is important to know whether and how HIIT and MICT regimens provide anti-inflammatory protection while modifying skeletal muscle bioenergetics and liver metabolism, especially in the aged animals of both sexes.

Even though two groups of male C57BL/6 J mice (5- and 24 months old), corresponding approximately to 30- and ~75-year-old humans^58^, were studied, only exercise protocols that matched the exercise tolerance of the older group were selected. An initial pilot experiment with age-matched animals, where VO_2_, VO_2_ max, and lactate levels were measured to estimate the maximal running capacity of the old group, provided preliminary results of the two training modalities selected for the study. Our results suggest that adult mice may need a more strenuous training in order to reap the health benefits of physical activity. Although most mouse or rat training studies are normally for 8+ weeks, we opted for a 4-week training, which may have been too short to elicit in adult animals some of the same adaptations noted in the old mice. It would have been of interest to use a middle-aged group of animals (12-months old) to determine whether the two types of exercise training confer similar benefits as those in the old group.

The observed metabolic crosstalk between muscle (cardiac and skeletal) and liver during aging^26,34^ and exercise regimes, shown in the present work, begs the important and unanswered question about the role of circadian modulation of exercise physiology in coordinating the physiological response to exercise training. Together with the intensity or duration of exercise training, differences in muscle function associated with circadian rhythms of, e.g., glycogen accumulation^61,62^, could underlie the changes in substrate utilization reported herein.

The well-known impact of exercise training in adipose tissue metabolism (e.g., increased lipolysis and greater FFA mobilization) was not investigated, thus the molecular underpinnings of exercise training in skeletal muscle transcriptome and liver metabolome of old MICT mice should be interpreted with caution because of the significant loss of body weight in old MICT mice *vs*. sedentary controls. To dissect the effects of exercise training from the generalized benefits of weight loss^63^, pair-feeding strategies will need to be implemented.

Although we do not have information on the longitudinal impact of exercise training initiated during adulthood, we surmise that prospective follow-up studies such as the Molecular Transducers of Physical Activity Consortium (MoTrPAC)^64^ and the Study of Longitudinal Aging in Mice (SLAM)^65^ will integrate multi-level omics data in an all-inclusive phenotypic investigation to unveil new mechanistic insights potentially translatable to humans^66,67^. Examples of multi-tissue omics analysis include a recent proteomics analysis in human participants in the GELSTALT study that revealed a top list of pathways potentially important in aging, such as the spliceosome^68^.

### Concluding remarks

An apparent genetic and metabolic reprogramming triggered by exercise training happens in mice subjected to moderate intensity, long-term exercise training. The reprogramming influences remodeling of energetic metabolism at the level of substrate fuel selection concomitantly with mitochondrial respiratory function as shown in skeletal muscle and liver while the serum metabolome suggested a broader organ participation. Global hierarchical clustering of the multiple variables measured from physiological, physical, and molecular levels further revealed the systemic impact of exercise training modalities in the two age groups analyzed. The salutary impact of exercise training was regimen- (MICT > HIIT) and age- (old > adult) dependent. Besides improvements in fasting blood glucose and overall physical fitness, the MICT regimen in old mice appears to remodel the liver cellular machinery related to lipid metabolism and gluconeogenesis such that sustained energy demand from the muscle is matched with increased glucose and other substrate fuels’ output from liver.

## Methods

### Animal model and study design

Male C57BL/6 J mice at either 5 months (adult group) or 24 months (old group) of age were obtained from the National Institute on Aging (NIA) Aged Rodent Colony (Charles Rivers Laboratories, Germantown, MD, USA). The mice were kept on a standard mouse diet (house chow 2018SX, Envigo, Frederick, MD, USA) with *ad libitum* access to food and water. Mice were single-housed in conventional micro-isolator cages (Lab Products, Seaford, DE, USA) on a light:dark 12:12-h schedule and maintained between 20–22 °C at 30–70% humidity. Mice were randomized to one of three experimental groups, namely sedentary control, HIIT, and MICT (*n* = 9 for each age group). The exercise protocols and study design are shown in Fig. 1a. Animal procedures, housing, and diets were in accordance with the guidelines issued by the Intramural Research Program of the National Institutes of Health (Animal Study Protocol 415-TGB-2018), and in compliance with all relevant ethical regulations regarding the care and use of research animals.

### Exercise protocols

Mice were trained daily (between 4:00–5:00 PM) for four consecutive weeks using an exercise treadmill (Columbus Instruments, Columbus, OH, USA) at an inclination of 30 ± 1 degrees. Two training protocols were tested: (1) HIIT, fueled mostly by creatine phosphate and stored carbohydrates in the form of glycogen, consisted of three short runs (2 min each) at 27 m/min with a one-min of active recovery (5 m/min) between bouts, and (2) MICT, an exercise training dependent on oxygen consumption and fueled mostly by stored fat, consisted of a single 45-min run at 13 m/min. The sedentary control mice were brought to the training room daily before the exercise groups and while in their home cage were exposed to the treadmill turned on in the background to ensure the same stress adaptation to the noise for all mice. Moreover, sedentary mice were restrained and manipulated daily to expose them to a comparable level of human interaction/handling as their exercised counterparts.

Treadmill work, expressed as J.day^−1^, was calculated as follows: Body weight (kg) × total vertical distance traveled (defined as treadmill speed (m/min) × % grade × Exercise time (min)).

### Body composition

At baseline and on the following day after the conclusion of the 4-week training session, mice were placed in the body comp clear plastic tube of a Bruker’s Minispec Whole Body Composition Analyzer LF90 (BRUKER, Billerica, MA, USA). This nuclear magnetic resonance device acquires and analyzes Time Domain-NMR signals from all protons in the entire sample volume and measures body fat, free body fluid, and lean tissue content. *n* = 9 mice per group.

### Glucose, lactate, and insulin determination and HOMA calculation

Thirty-six hours after the last bout of training, mice were fasted for 3 h (8:00 AM–11:00 AM) prior to the collection of tissues and blood at sacrifice. Fasting blood glucose (FBG) was measured in whole blood using Breeze2 handheld glucometer (Bayer, Mishawaka, IN, USA). A single drop of blood was required for the measure of lactate using Lactate Plus Meter (NOVA Biomedical, Waltham, MA, USA). Serum insulin was measured using the Ultra-Sensitive Mouse Insulin ELISA kit (Crystal Chem, Downers Grove, IL, USA) according to the manufacturer’s protocol. Insulin resistance was calculated from fasting glucose and insulin values using the HOMA2-IR calculator software available from the Oxford Centre for Diabetes, Endocrinology and Metabolism, Diabetes Trials Unit website (http://www.dtu.ox.ac.uk). *n* = 9 mice per group.

### In vivo metabolic assessment

At the conclusion of the 4-week training period, the metabolic rate of each mouse was assessed by indirect calorimetry in open-circuit Oxymax chambers using the Comprehensive Lab Animal Monitoring System (CLAMS; Columbus Instruments, Columbus, OH, USA). Mice were single housed with *ad libitum* access to food and water and maintained at 20–22 °C under a 12:12-h light:dark cycle (light period 0600–1800). All mice were acclimated to monitoring cages for 12 h prior to recording. Sample air was dried and passed through an oxygen sensor for determination of oxygen content. Oxygen consumption was determined by measuring oxygen concentration in air entering the chamber compared with air leaving the chamber. The sensor was calibrated against a standard gas mix containing defined quantities of oxygen, carbon dioxide and nitrogen. Constant airflow (0.6 L/min) was drawn through the chamber and monitored by a mass-sensitive flow meter. The concentrations of oxygen and carbon dioxide were monitored at the inlet and outlet of the sealed chambers to calculate oxygen consumption. Measurement in each chamber was recorded for 30 s at 30-min intervals for a total of 60 h. Ambulatory activity (both horizontal and vertical) was also monitored. *n* = 9 mice per group.

### Assessment of muscle function/motor behavior

At the conclusion of the 4-week training period, each mouse was tested for forelimb strength using two tests: (1) *Grip Strength* using the Grip Strength Meter (Columbus Instruments), at the completion of five measurements, the highest and lowest values were discarded and the remaining three were kept for analysis; (2) *Wire hang*, the test consisted of measuring the time before the mouse falls from a hanging wire, and is considered complete if the mouse is still hanging after 60 s. *n* = 9 mice per group.

### Tissue collection and histology

After collection of physiological measurements, mice were let to rest for 12 h and were then euthanized by cervical dislocation. A midline laparotomy was performed, and blood (up to 1 mL) was collected from the inferior vena cava. Brain, lungs, heart, liver, kidneys, spleen, pancreas, adipose tissue, and skeletal muscle (gastrocnemius, quadriceps, and soleus) were excised, and snap-frozen in liquid nitrogen for storage.

For histological analysis, gastrocnemius muscle was quickly dissected and fixed in a block using Tissue Tek (VWR, Radnor, PA, USA) and isopentane (Sigma-Aldrich, St-Louis, MO, USA). After fixation, samples were stored at -80 °C.

### Electron microscope analysis

After dissection, samples from gastrocnemius were quickly washed in 0.1 M cacodylate buffer (pH 7) and fixed in a mixture of 2.5% glutaraldehyde—2% paraformaldehyde in the same buffer for 1 h at room temperature and overnight at 4 °C. Samples were then washed in buffer and post-fixed in 1% in osmium tetroxide in buffer for 1 h. After washing, the specimens were dehydrated in an ethanol series, embedded in epoxy resin (Embed-812, EMS, USA), and the samples were positioned in the molds to get cross-sections of the fibers.

Thin sections (40–60 nm width) were obtained in an Ultracut Reicher ultramicrotome and stained with uranyl acetate and lead citrate^69^. The sections were viewed and photographed in a Jeol Jem 1400 electron microscope at the Servicio Centralizado de Apoyo a la Investigación (SCAI; University of Córdoba, Spain). From this material, pictures were taken at 25,000X magnification from different parts of the fibers: peripheral (with a visible endomysium) and internal areas. Six to eight pictures per cell from 10–12 cells per animal (4–5 animals per experimental group) were captured. In some cases, pictures at lower magnification allowed us to get wider perspectives of the fiber ultrastructure. Several quantitative analyses were performed as follows:

1. Measurement of the reference cell area (e.g., the total area of the fiber included in the picture excluding endomysium, if present) and the area of all the mitochondria included in the picture. From this analysis, mitochondrial density expressed as “mitochondrial fractional area” (Fa) and as “mitochondrial number” (Na) was determined. Fa represents the area fraction of mitochondria, e.g., the summed area of all mitochondria divided by the reference area in a given image, whereas Na is the number of mitochondria located in a reference area.

2. Mitochondrial planimetric parameters were measured from these pictures by focusing on mitochondrial area and circularity. All these procedures were carried out using ImageJ software (NIH, Bethesda, MD, USA).

### Determination of ETC activities

Activities of NADH:coenzyme Q1 oxidoreductase (complex I), succinate dehydrogenase (complex II), ubiquinol:cytochrome c oxidoreductase (complex III), NADH:cytochrome c reductase (complex I–III), succinate:cytochrome c reductase (complex II–III), cytochrome c oxidase (complex IV), and citrate synthase (CS) were determined in gastrocnemius skeletal muscle from mice by spectrophotometric assays^70^. All activities were assayed using 20 µL of muscle homogenate (diluted to 2 mg protein /ml) in 880 µL of reaction medium. Specific activities are expressed as nmoles.min^−1^.mg^−1^ protein. In brief,

**Complex I** was assayed in a reaction medium containing 50 mM potassium phosphate buffer, pH 7.5, 60 μM ubiquinone_1_, 300 μM potassium cyanide, 3 mg/ml bovine serum albumin (BSA), and 100 μM NADH. Reaction was carried out at 340 nm at 37 °C. Specific activity is the result of subtracting the unspecific, non-complex I activity measured with 10 μM rotenone, from the total NADH:ubiquinone oxidoreductase activity (without rotenone). The extinction coefficient for NADH (340 nm) is *ε* = 6.2 mmol^−1^cm^−1^.

**Complex II** was assayed in a reaction medium containing 25 mM potassium phosphate buffer, pH 7.5, 20 mM sodium succinate (substrate), 80 μM 2,6-dichlorophenolindophenol (DCPIP, artificial electron acceptor), 50 μM decylubiquinone, 300 μM potassium cyanide, and 1 mg/ml BSA. The specific activity is the result of subtracting the baseline (without decylubiquinone) from the total succinate:ubiquinone oxidoreductase activity with and without 10 mM malonate as specific inhibitor. The extinction coefficient for DCPIP (600 nm) is *ε* = 19.1 mmol^−1^cm^−1^.

**Complex III** was assayed in a reaction medium containing 25 mM potassium phosphate buffer, pH 7.5, 75 μM cytochrome c, 100 μM decylubiquinol, 500 μM potassium cyanide, 100 μM EDTA, and 0.025% Tween 20 (v/v). To determine the specificity of the reaction, 10 μg/ml antimycin A was used. The specific activity is the result of subtracting the antimycin A-resistant activity from the total decylubiquinol cytochrome c oxidoreductase activity. The extinction coefficient for cytochrome c (550 nm) is *ε* = 18.5 mmol^−1^cm^−1^.

**Complex IV** was assayed in a reaction medium containing 50 mM potassium phosphate buffer, pH 7.0, and 60 μM reduced cytochrome c. The specific activity is the result of subtracting the potassium cyanide (300 μM)-resistant activity from the total cytochrome c oxidase activity. The extinction coefficient for cytochrome c (550 nm) is *ε* = 18.5 mmol^−1^cm^−1^.

**Complex I** + **III** was assayed in a reaction medium containing 50 mM potassium phosphate buffer, pH 7.5, 50 μM cytochrome c, 200 μM NADH, 1 mg/ml BSA, and 300 μM potassium cyanide. The specific activity is the result of subtracting the unspecific activity measured with 10 μM rotenone from the total NADH:cytochrome c oxidoreductase activity. The extinction coefficient for cytochrome c (550 nm) is *ε* = 18.5 mmol^−1^cm^−1^

**Complex II** + **III** was assayed in a reaction medium containing 20 mM potassium phosphate buffer, pH 7.5, 300 μM potassium cyanide, 50 μM cytochrome c, and 10 mM sodium succinate. To determine the specificity of the reaction, 10 mΜ malonate was used as inhibitor. The extinction coefficient of the cytochrome c (550 nm) is *ε* = 18.5 mmol^−1^cm^−1^.

**CS** enzyme activity was assayed in a reaction medium containing 100 mM Tris-HCl, pH 8.0, 100 μM 5,5-dithio-bis-(2-nitrobenzoic acid) (DTNB), 300 μM acetyl-CoA, 0.1% Triton X-100 (v/v), and 500 μM oxalacetate. The extinction coefficient for DTNB (412 nm) is *ε* = 13.6 mmol^−1^cm^−1^.

### Measurements of total and redox state of CoQ

CoQ was extracted from quadricep muscle tissues after addition of 4 ml hexane/ethanol mixture (5/2, v/v) and vortexed for 2 min. The mixture was centrifuged at 1000 × *g* for 5 min at room temperature, after which the upper phase was carefully transferred into a 20-ml glass scintillation vial and the extraction procedure repeated once more. The combined extract was evaporated under a gentle stream of nitrogen and the residue was dissolved in 0.1 ml of n-propanol. A 50 µl aliquot of the reconstituted extract was directly injected into a HPLC system with a C18 reversed-phase column (15 cm, Phenomenex) and a Coulochem III electrochemical detector (ECD).

The redox state of CoQ was determined in single sample that was processed each time to prevent artificial oxidation of ubiquinol. Lipids were extracted by adding 300 µl of 2-propanol and 1:1000 of 2-mercaptoethanol to 100 µl of homogenate. Samples were centrifuged at 16,000 × *g* for 1 min at 4 °C and 100 µl of the supernatant was immediately injected into a 166–126 HPLC system (Beckman-Coulter) equipped with an UV/Vis detector (System Gold R 168, Beckman-Coulter) and an electrochemical detector (Coulochem III ESA). Separation was carried out in a 15 cm Kromasil C18 column (Scharlab, Spain) at 40 °C with a mobile phase of methanol/n-propanol (65:35) containing 1.42 mM lithium perchlorate at a flow rate of 1 ml/min. UV spectrum was used to identify the different forms of ubiquinone (oxidized CoQ_9_ and CoQ_10_ with maximum absorption at 275 nm) and ubiquinol (reduced CoQ, CoQ_9_H_2_, and CoQ_10_H_2_, with maximum absorption at 290 nm) using specific standards, and the electrochemical readings were used for their quantification.

### Protein extraction, gel electrophoresis, and western blot analysis

Muscle protein extracts were prepared by adding 50 mg frozen tissue to 1 mL of radioimmunoprecipitation buffer (Boston BioProducts, Boston, MA, USA) containing 50 mM Tris-HCl, 150 mM NaCl, 1% NP-40, 0.5% sodium deoxycholate, and 0.1% SDS, pH 7.4 ± 0.2, and supplemented with commercially available cocktails of phosphatase and protease inhibitors (Sigma-Aldrich), along with the deacetylase inhibitors nicotinamide, sodium butyrate and trichostatin-A (Sigma-Aldrich) (Supplementary Table 9). Following homogenization using TissueLyser II (Qiagen, Germantown, MD, USA), samples were centrifuged (16,000 × *g*, 30 min at 4 °C) and protein concentration in clarified lysates was determined using the Bradford assay (Bio-Rad, Carlsbad, CA, USA). Equal amounts (10 ug) of each sample were separated on 26-wells 4–15% Criterion TGX precast gels (Bio-Rad) with Laemmli buffer under reducing conditions and then electrotransferred to ImmunoBlot nitrocellulose membranes using the Trans-Blot Turbo Transfer System from Bio-Rad. After membrane staining with Ponceau S solution (#P7170, Sigma-Aldrich), Western blot was performed according to standard procedures, which involved a blocking step in PBS/0.1% Tween 20 supplemented with either 5% non-fat milk or 3% bovine serum albumin (Sigma-Aldrich) and incubation with several commercially available primary antibodies listed in Supplementary Table 10. Bound antibodies were detected with HRP-conjugated secondary antibodies (Santa Cruz Biotechnology, Dallas, TX, USA) and visualized using Amersham ECL Prime Western Blotting Detection Reagent chemiluminescence kit (GE Healthcare, Laurel, MD, USA). Imaging of the signal was captured with Amersham Imager 600 (GE Healthcare, Piscataway, NJ, USA). Quantification of the protein bands was performed by volume densitometry using ImageJ software (National Institutes of Health, Bethesda, MD, USA) and normalization to Ponceau-stained images of the membranes. All blots or gels were derived from the same experiment and they were processed in parallel.

### Microarray analysis

Gastrocnemius RNA was isolated using a RNeasy Mini kit (Qiagen, Germantown, MD, USA). Total RNA quantity and quality were evaluated using the Bioanalyzer RNA 6000 Chip (Agilent Technologies, Santa Clara, CA, USA). Cy3-labeled cRNA was prepared from 200 ng of total RNA using the Agilent Low-Input Quick Amp Labeling Kit and was purified and quantitated according to the manufacturer’s instructions. A total of 300 ng Cy3-labeled cRNA was hybridized to Agilent SurePrint G3 Mouse GE 8X60K microarrays (G4852A) for 17 h at 65 °C. After post-hybridization rinses, arrays were scanned using an Agilent SureScan Microarray Scanner at 5 μm resolution, and hybridization intensity data were extracted from the scanned images using Agilent’s Feature Extraction Software 9.1. Raw data were submitted to *Z*-normalization, and principal component analysis (PCA) was performed on the normalized *Z*-scores of all detectable probes in the samples. Significant genes were selected by the *z*-test < 0.05, false-discovery rate < 0.30, *z*-ratio > 1.5 in either direction and ANOVA *P*-value < 0.05. Parametric analysis of gene set enrichment (PAGE) was then carried out to detect significantly altered gene sets^71^. *n* = 4 per group.

### Quantitative real-time PCR

Total RNA was isolated from gastrocnemius muscle using the Trizol reagent (Invitrogen). Complementary DNA was reverse-transcribed using iScript reverse-transcription supermix (Bio-Rad). The real-time PCR was performed on individual cDNAs by using SYBR® Green PCR master mix in a Quantstudio 7 Flex Real-time PCR system (Applied Biosystems) to measure duplex DNA formation. The calculation of mRNA expression was performed by the 2 − ^ΔΔCT^ method normalized to the expression of β-actin and Rn18S. The oligonucleotide primer sequences are found in Supplementary Table 11; *n* = 4 per group.

### Cytokine analysis by multiplex ELISA

Protein loading concentrations (75 or 100 µg/well) were assessed in duplicate on a 9-plex Mesoscale Discovery V-PLEX Proinflammatory Panel 1 (Cat # K15048D) for mouse, using the protocol suggested by the manufacturer. *n* = 5 per group.

### Metabolomics analysis

Metabolomic analysis was performed by the West Coast Metabolomics Center at UC Davis (Davis, CA) in liver and serum of 3-h fasted animals. As we briefly reported^34,48,72^, liver and serum were extracted in an acetonitrile:isopropanol:water (3:3:2) solution, vortexed, centrifuged, and the supernatants aliquoted for downstream analysis. After a series of evaporation and reconstitution steps in 50% acetonitrile, internal standards (C08-C30, fatty acid methyl esters) were added to the dried sample, which was then derivatized for trimethylsilylation of acidic protons. Data were acquired using a method developed in the Fiehn laboratory^73^ and applied by Mitchell et al.^48^. In brief, metabolites were measured using a rtx5Sil-MS column (made of 95% dimethyl, 5% diphenyl-polysiloxane coated on fused silica; Restek Corporation; Bellefonte PA) protected by an empty guard column. This chromatography method yields excellent retention and separation of primary metabolite classes (amino acids, hydroxyl acids, carbohydrates, sugar acids, sterols, aromatics, nucleosides, amines, and miscellaneous compounds) with arrow peak widths of 2–3 s and very good within-series retention time reproducibility of better than 0.2 s absolute deviation of retention times. The mobile phase consisted of helium, with a flow rate of 1 mL/min, and injection volume of 0.5 μL. The following mass spectrometry parameters were used: a Leco Pegasus IV mass spectrometer with unit mass resolution at 17 spectra s-1 from 80–500 Da at −70 eV for elution of metabolites. As a quality control, for each sequence of sample extractions, one blank negative control was performed by applying the total procedure (e.g., all materials and plastic ware) without biological sample. Result files were transformed by calculating the sum intensities of all structurally identified compounds for each sample, and subsequently dividing all data associated with a sample by the corresponding metabolite sum. The resulting data were multiplied by a constant factor in order to obtain values without decimal places. Intensities of identified metabolites with more than one peak (e.g., for the syn- and anti-forms of methoximated reducing sugars) were summed to only one value in the transformed data set. The original non-transformed data set was retained. Relative metabolite levels represent the MS peak amplitude normalized with respect to the total metabolites returned, but disregarding unknowns that might potentially comprise artifact peaks or chemical contaminants. *n* = 9 per group.

### Hierarchical clustering

Physiological, biochemical, and serum metabolomics data were Z-score normalized across the six experimental groups. Unsupervised hierarchical clustering was performed on the normalized data with the Polar Heatmap with dendogram v1.0 from Origin 2021b (Origin Lab Corp., Northampton, MA, USA) using average linkage and uncentered similarity metrics. Principal component analysis was performed with the same input data using the multivariate statistics subroutine of Origin 2021b.

### Statistical analysis

No statistical methods were used to predetermine sample size. Investigators were not blinded to allocation during experiments and outcome assessment. GraphPad Prism 7 was used to determine whether data sets passed the D’Agostino & Pearson normality test (*α* = 0.05). Generally, data were expressed as box-and whisker plots, with the difference between sedentary, HIIT, and MICT in each age group assessed by one-way ANOVA or Kruskal–Wallis H test with Tukey’s post-hoc tests, with *p* ≤ 0.05 considered statistically significant. In other instances, scatter plots depicting the coordinates of each mouse were presented with mean ± SEM. From the TEM stereological analysis of the gastrocnemius, the comparison between groups was performed using regular two-way ANOVA with Sidak’s post-hoc tests by considering the two independent factors: age (adult or old), type of exercise training (none, HIIT, or MICT), and their interaction. A *p* ≤ 0.05 was considered statistically significant. Analyses were performed using GraphPad Prism 7.0 (San Diego, CA, USA) and Excel 2018 (Microsoft Corp., Redmond, WA, USA).

## Supplementary information

Supplementary material

## Data Availability

The data sets generated during and/or analyzed during the current study are available from the corresponding author, Rafael de Cabo (decabora@mail.nih.gov), on reasonable request. Processed gene expression data can be obtained from Gene Expression Omnibus (GEO) (GEO: GSE175622). Mass spectrometry metabolomic data have been deposited in the Metabolomics Workbench (http://www.metabolomicsworkbench.org) with project identifier PR001142, 10.21228/M8368X.
