# Supplementary material for: Age-dependent impact of two exercise training regimens on genomic and metabolic remodeling in skeletal muscle and liver of male mice
Source: NPJ Aging. 2022 Jun 27;8(1):8. doi: 10.1038/s41514-022-00089-8 (PMC9237062; doi:10.1038/s41514-022-00089-8)
Supplement: Supplementary file 1 — Supplementary material [file 41514_2022_89_MOESM1_ESM.pdf]

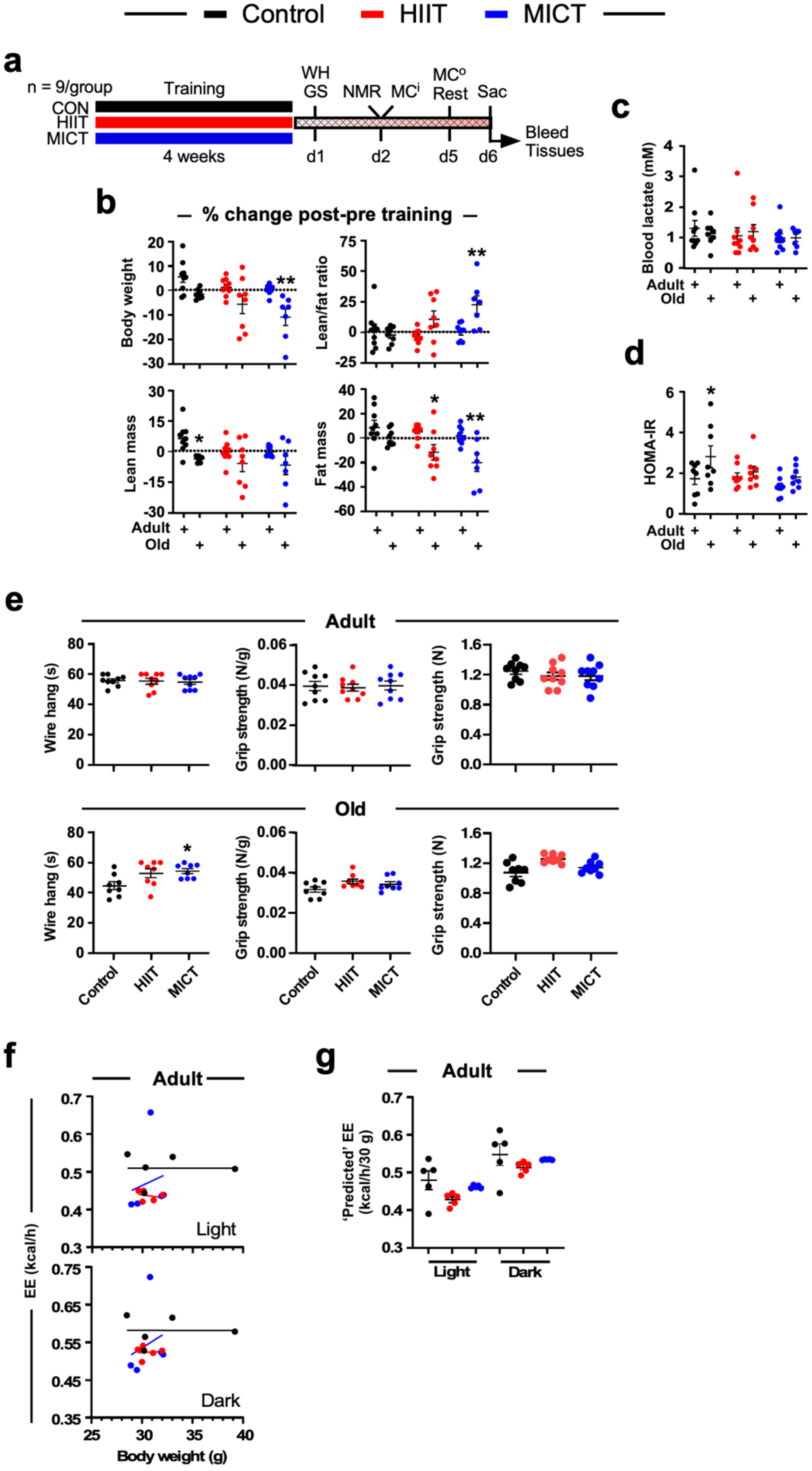

Control
HIIT
MICT
a
n = 9/group
Training
CON
HIIT
MICT
4 weeks
WH
GS
NMR
MCi
MCo
Rest
Sac
d1
d2
d5
d6
Bleed
Tissues
b
% change post-pre training
Body weight
Lean/fat ratio
Lean mass
Fat mass
Adult
Old
c
Blood lactate (mM)
d
HOMA-IR
e
Adult
Old
Wire hang (s)
Grip strength (N/g)
Grip strength (N)
Control
HIIT
MICT
f
Adult
Light
Dark
EE (kcal/h)
Body weight (g)
g
Adult
'Predicted' EE (kcal/h/30 g)
Light
Dark

Supplementary Figure 1. Impact of a 4-week exercise training on physiological parameters in adult and old male mice. a Workflow chart that describes the handling procedure for each cohort. Abbreviations: WH, wire hang test; GS, grip strength test; NMR, body weight and body composition; MC^i, entry into the metabolic cage, MC^o, exit of the metabolic cage after 72 h. **b** Percent change from baseline in body weight, lean mass, fat mass and lean-to-fat ratio. n = 7-9 mice per group. **c** Blood lactate level, n = 7-9 mice per group. **d** HOMA-IR index. n = 8 mice per group. **e** Forepaw grip strength was assessed at the conclusion of the training period, n = 9 mice per group. **f** Sedentary and exercised adult mice were placed in metabolic cages to measure several metabolic parameters, including energy expenditure (EE) over 60 hours, as detailed in the Method section. Nonsignificant relationship between EE and body mass in adult sedentary and exercised mice during the light and dark phases, n = 5 mice per group. The regression line equations can be found in Supplementary Table 2. **g** Lack of significant effect of exercise on EE in adult mice from the ANCOVA analysis at a given body mass of 30 g; n= 5 mice per group. Data in panels **b-d** have been analyzed using two-way ANOVA with the Sidak post-hoc test. *, ** $p \leq 0.05$ and ≤ 0.01 in old vs. adult, respectively. Related to Fig. 1.

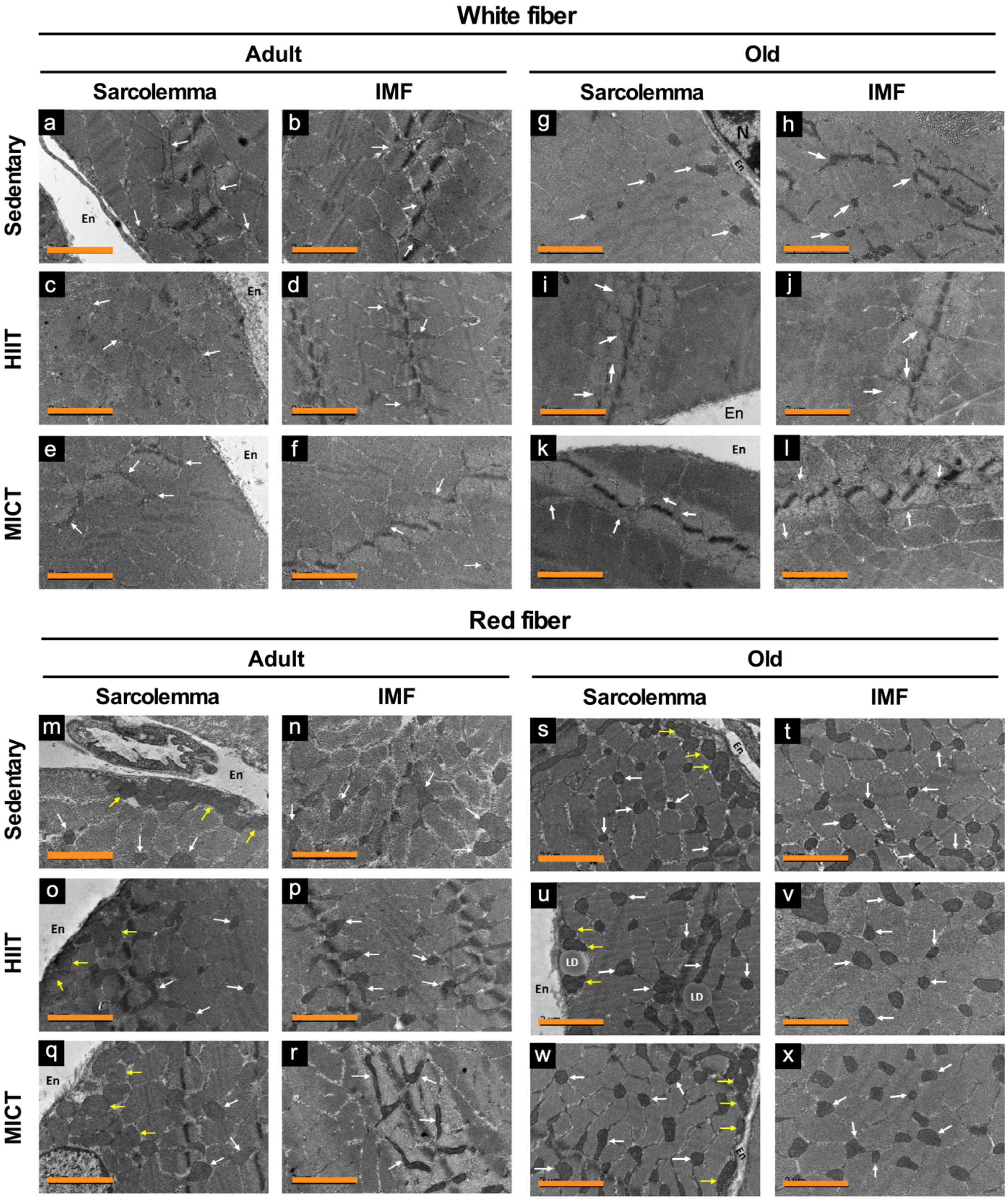

Supplementary Figure 2. Transmission electron microscopy (TEM) study of skeletal muscle fibers from adult and old mice. a-l Cross-sectioned white muscle fibers from adult (**a-f**) and old (**g-l**) mice. The mice were either sedentary (**a, b, g, h**) or subjected to 4-week daily training exercise, HIIT (**c, d, i, j**) or MICT (**e, f, k, l**). Shown are the sarcolemmal region of the muscle fiber with visible endomysium (En) (adult, **a, c, e**; old, **g, i, k**) and the intermyofibrillar area (adult, **b, d, f**; old, **h, j, l**). Mitochondria are marked

with white arrows. **m-x** Cross-sectioned red muscle fibers from adult (**m-r**) and old (**s-x**) mice. The mice were either sedentary (**m, n, s, t**) or subjected to 4-week daily training exercise, HIIT (**o, p, u, v**) or MICT (**q, r, w, x**). Shown are the sarcolemmal region of the muscle fiber with visible endomysium (En) (adult, **m, o, q**; old, **s, u, w**) and the intermyofibrillar area (adult, **n, p, r**; old, **t, v, x**). Subsarcolemmal mitochondria are indicated with yellow arrows and intermyofibrillar mitochondria with white arrows. Scale bar, 2 μm. Related to Fig. 2 and Supplementary Fig. 3. The enlarged versions of all 24 panels (**a-x**) have been deposited as a Source data file.

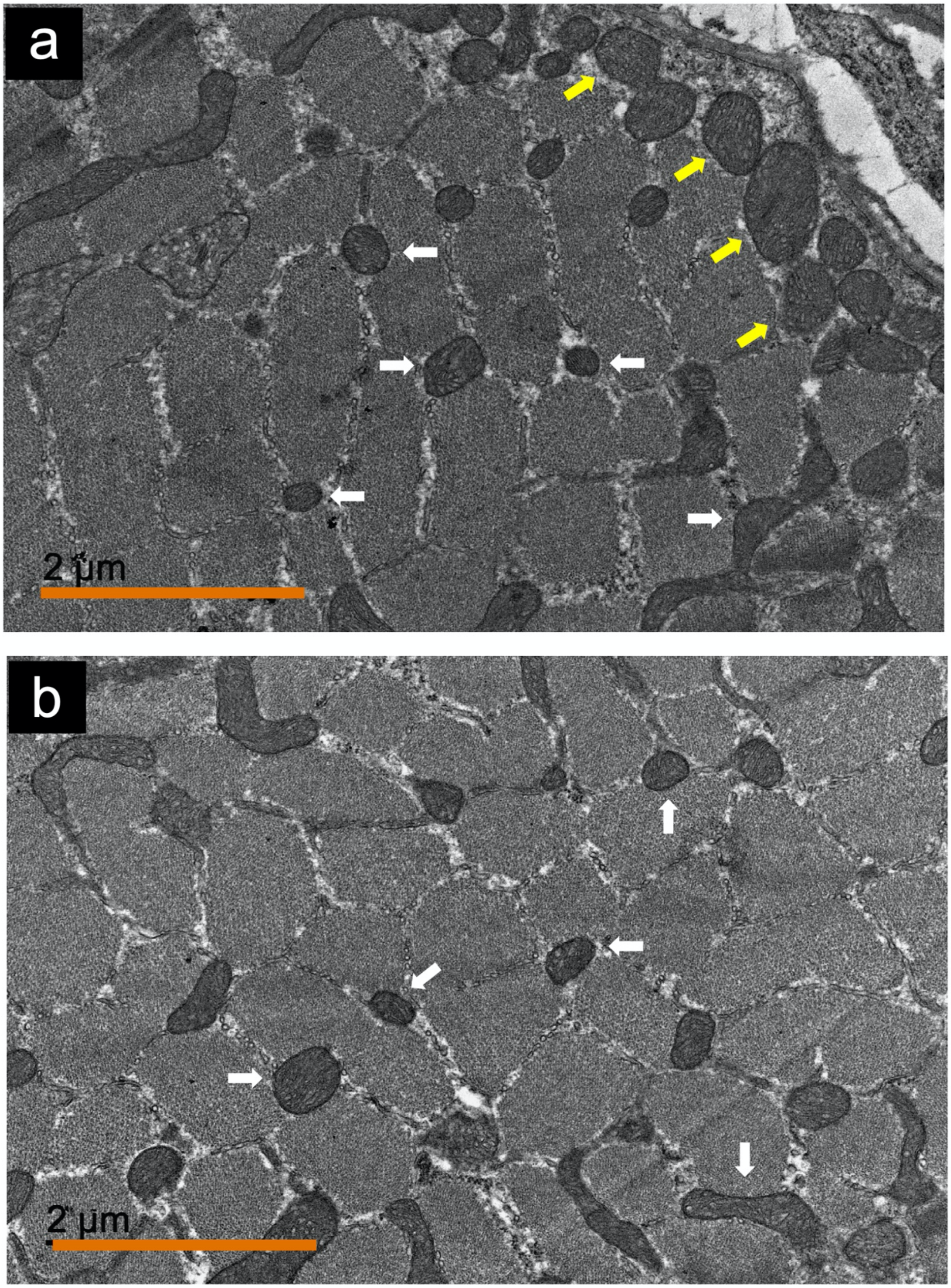

Supplementary Figure 3. Representative EM micrographs of red muscle fibers from sedentary old mice. a, b Enlargement of panels **s** and **t** from Supplementary Fig. 2 is depicted. These representative images were taken at 25,000 X, a magnification that enables evaluation of mitochondrial morphology and allows for exact planimetric analysis of the mitochondria (e.g., area and circularity), and at the same time contains a cell portion large enough to determine the ‘fractional area” and the “numerical density profile”.

Many photos were obtained of each fiber, both from the internal portions and peripheral areas, allowing a fairly accurate estimate for the entire fiber. Subsarcolemmal mitochondria are indicated with yellow arrows and intermyofibrillar mitochondria with white arrows. Scale bar, 2 μm. Related to Fig. 2, Supplementary Fig. 2, and Supplementary Fig. 4.

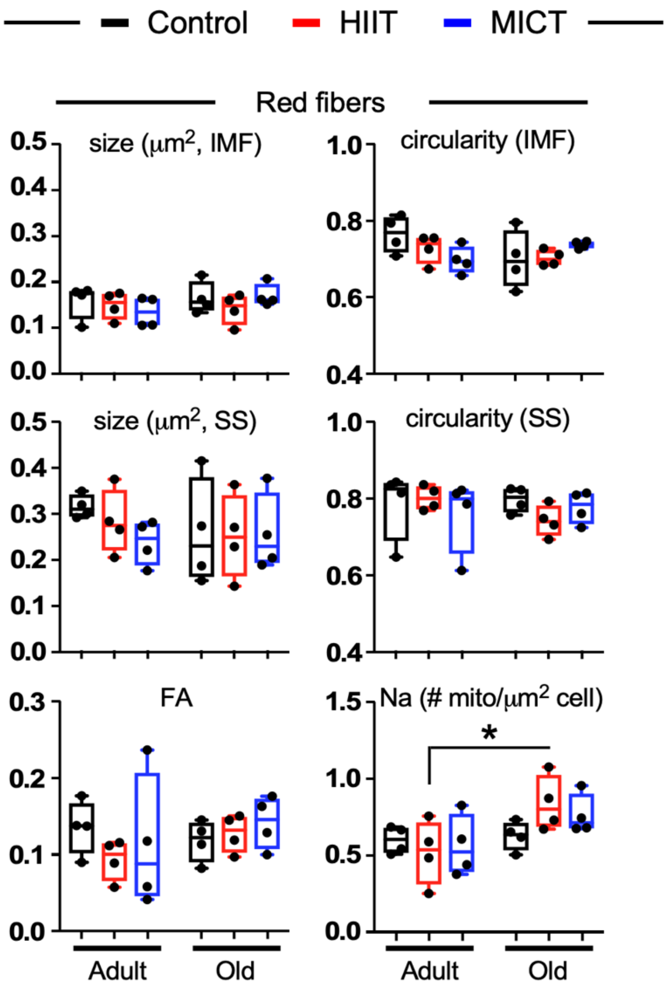

Supplementary Figure 4. Morphometric and stereological assessment of mitochondria in red muscle fibers of adult and old mice. Mitochondrial size and circularity of intermyofibrillar mitochondria (IMF) (upper panels) and subsarcolemmal mitochondria (SS) (middle panels) from adult and old sedentary mice or mice subjected to a 4-week daily exercise regimen, HIIT or MICT. Fractional area (FA) of mitochondria and numerical density profiles are depicted in the lower panels. Results are presented as box and whisker plots, with minimum, lower quartile (Q1), median (Q2), upper quartile (Q3), and maximum values. n=4 mice per group. *, $p \leq 0.05$ by two-way ANOVA with the Sidak post-hoc test. Related to Fig. 2.

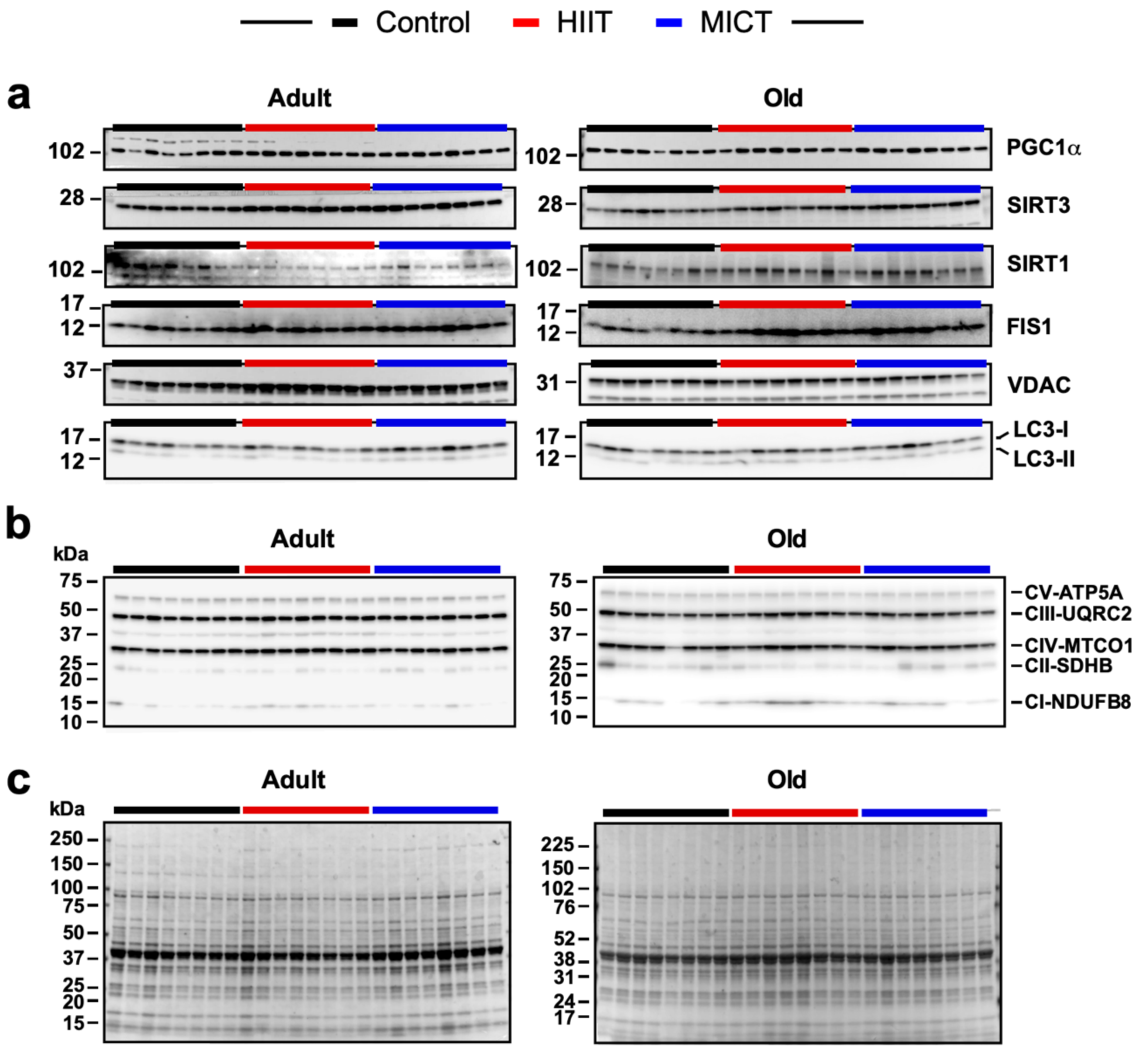

Supplementary Figure 5. Impact of exercise training on key bioenergetic pathways in skeletal muscle. **a** Immunoblots for PGC-1α, SIRT3, SIRT1, FIS1, VDAC and LC3 proteins from gastrocnemius muscle homogenates of adult (5-mo old) and old (24-mo old) mice that were either sedentary or subjected to HIIT or MICT for 4 weeks. n = 8 mice per group. The uncropped figures can be found in the Source Data file. **b** Expression levels of the multi-protein *OXPHOS* complexes by immunoblotting. **c** Ponceau S staining of representative membranes. The molecular mass of protein standards (in kDa) is shown on the left. Related to Fig. 3.

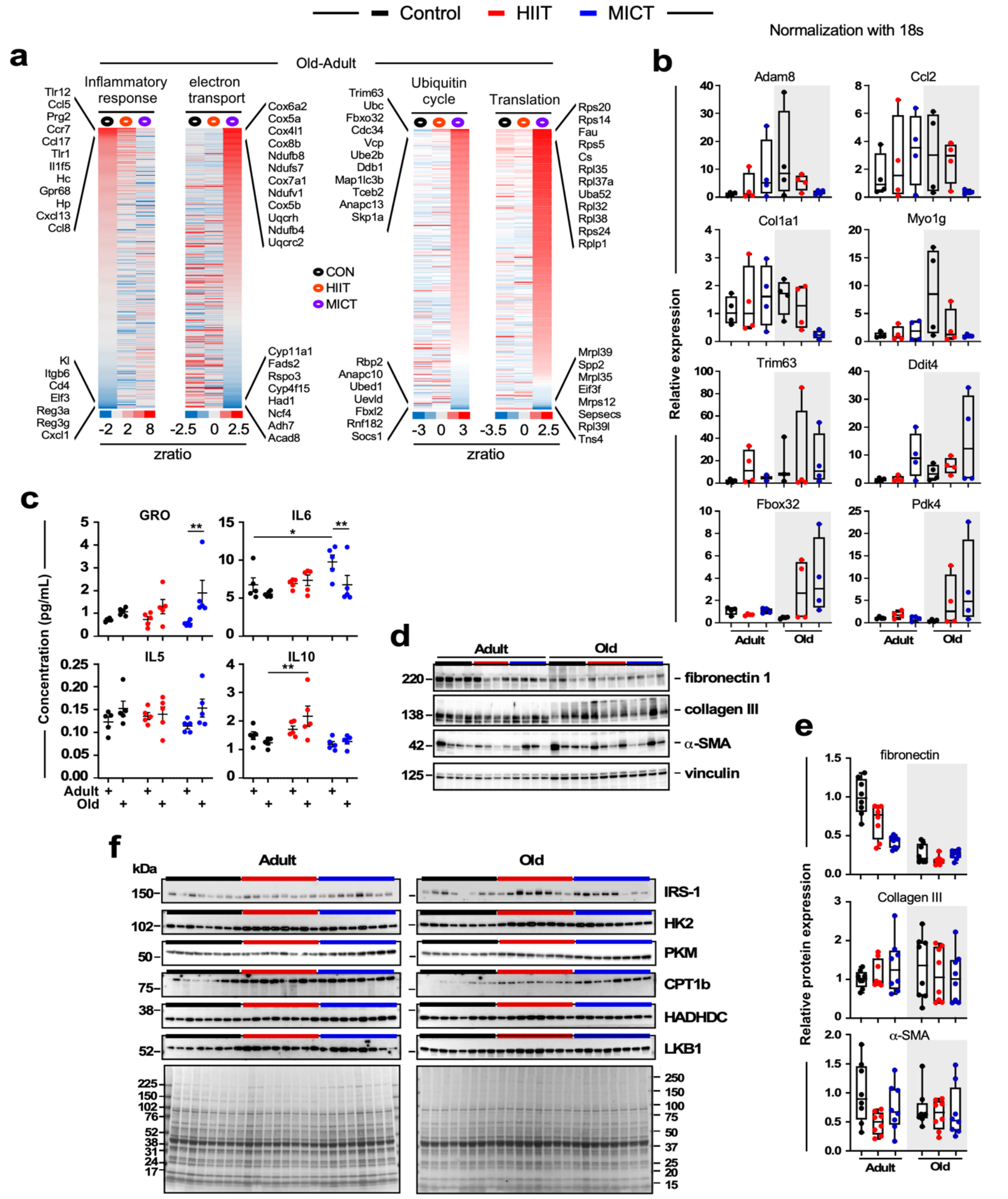

Supplementary Figure 6. Impact of exercise on molecular determinants from skeletal muscle of adult and old mice. a Heatmap visualization of the effect of exercise training on the expression of genes enriched in the indicated biological processes between old and adult mice. **b** Quantitative real-time PCR of 8 target genes in skeletal muscle tissues. Results are depicted as box and whisker plots, with minimum, lower

quartile (Q1), median (Q2), upper quartile (Q3), and maximum values. n = 4 mice per group. **c** Levels of GRO/CXCL1, IL5, IL6, and IL10 in skeletal muscle extracts. Date is presented as mean ± SEM, n = 5 mice per group. * $p \leq 0.05$, ** $p \leq 0.01$, by two-way ANOVA with the Sidak post-hoc test. **d** Western blot for the indicated proteins in skeletal muscle extracts. **e** Densitometric quantification after normalization with vinculin levels. Results are depicted as box and whisker plots, with minimum, lower quartile (Q1), median (Q2), upper quartile (Q3), and maximum values. n = 8 per group. **f** Muscle extracts were immunoblotted as in **d** with molecular mass markers (kDa) on the left, n = 8 mice per group. Ponceau S staining of representative membranes in the bottom panels. The uncropped images can be found in Source data file. Related to Fig. 4.

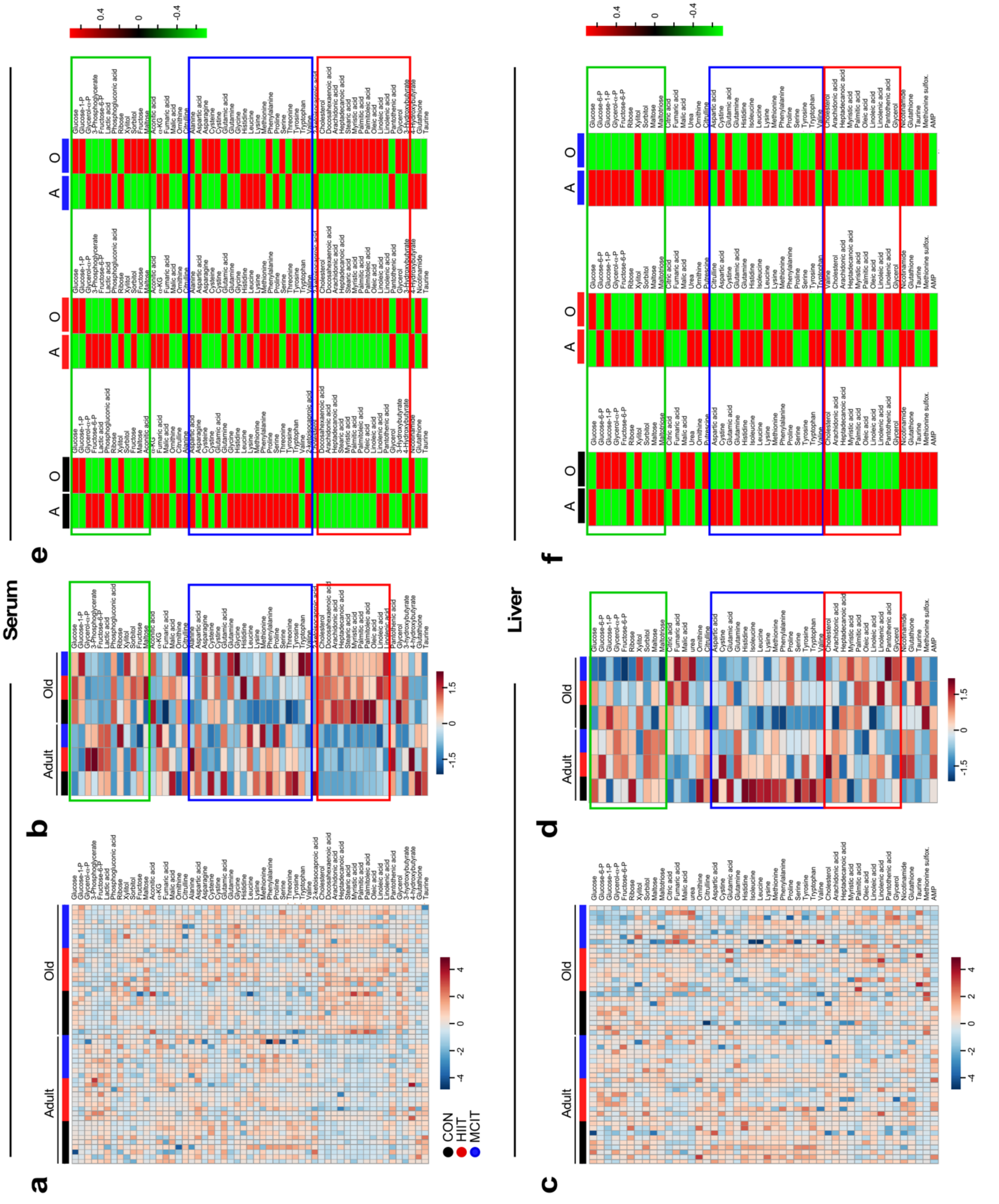

Supplementary Figure 7. Heatmaps depicting levels of modulated metabolites and associated pathways in serum and liver from sedentary (CON) and exercised adult and old mice. a and **c** Heatmaps depicting the metabolites that account for the separation between CON, HIIT and MICT

metabolic profiles from the PLS-DA analysis. Each row represents a single metabolite, and each column depicts a single mouse (n = 9 mice per experimental group). There were 55 and 46 metabolites enriched in serum (**a**) and liver (**c**), respectively. Black bar, CON; red bar, HIIT; blue bar, MICT. For each metabolite, the type (positive or negative) and strength (color intensity) of abundance are coded red and blue, respectively, normalized between 4 and -4 according to the bar on the bottom. **b** and **d** Heatmaps depicting average values of the metabolites enriched in serum (**b**) and liver (**d**) as a function of exercise. Boxed areas depict cluster of metabolites. Green, glucose catabolism; blue, amino acids; red, lipids, cholesterol and glycerol. For each metabolite, the type (positive or negative) and strength (color intensity) of relative abundance are coded red and blue, respectively, normalized between 1.5 and -1.5 according to the bar on the bottom. **e** and **f** Heatmaps depicting average values of the 46 metabolites enriched in adult (A) and old (O) mice that remained sedentary (left panels) or subjected to HIIT (middle panels) or MICT (right panels). Boxed areas depict cluster of metabolites. Green, glucose catabolism; blue, amino acids; red, lipids, cholesterol and glycerol. The pseudocolor scaling of the standardized expression is from low (green) to high (red), normalized between -0.6 and 0.6 according to the bar on the right. Related to Fig. 5 and 6.

Supplementary Fig. 8. Effect of age, exercise regimen, and interaction 'age x exercise' on the serum and liver metabolome in mice. The metabolites with an asterisk are those that do not show significant changes either with age or treatment or interaction.

	Serum				Liver			
	Metabolite	Age	Treatment	Interaction	Metabolite	Age	Treatment	Interaction
Glucose catabolism	Glucose	** (18.4%)	NS	NS	Glucose	** (15.4%)	NS	** (14.8%)
	Glucose-6-P	** (19.3%)	NS	NS	Glucose-6-P	** (10%)	** (14.8%)	*** (20.3)
	Glucose-1-P	**** (38.3%)	NS	NS	Glucose-1-P	NS	NS	** (21.6%)
	Glycerol-a-P	**** (49.8%)	NS	*(6.26%)	Glycerol-a-P	*(6.93%)	NS	** (18.2%)
	3-Phosphoglycerate	**** (25.7%)	NS	*(7.76%)	3-Phosphoglycerate	NS	NS	NS
	Fructose-6-P	**** (62.3%)	NS	NS	Fructose-6-P	*(7.95%)	*(13.7%)	** (16.1%)
	Lactic acid	**** (66.5%)	NS	NS	Lactic acid	NS	NS	NS
	Phosphogluconic acid	**** (49%)	NS	NS	Phosphogluconic acid*	NS	NS	NS
	Ribose	*** (20.8%)	NS	NS	Ribose	*** (19.4%)	NS	NS
	Xylitol	*** (23%)	NS	NS	Xylitol	**** (34.5%)	NS	NS
	Sorbitol	NS	*(11.5%)	NS	Sorbitol	*** (25%)	NS	NS
	Fructose	** (17.4%)	*(9.64%)	NS	Fructose*	NS	NS	NS
	Maltose	NS	*** (26.7%)	*(13.3%)	Maltose	** (15.5%)	NS	NS
	Maltotriose*	NS	NS	NS	Maltotriose	*(10.1%)	NS	*(12.4%)
TCA cycle intermediates	Citric acid*	NS	NS	NS	Citric acid	*(8.57%)	NS	NS
	Aconitic acid	*** (20.7%)	*(11.5%)	*(9.23%)	Aconitic acid*	NS	NS	NS
	Isocitric acid*	NS	NS	NS	Isocitric acid*	NS	NS	NS
	a-KG	**** (61.4%)	NS	NS	a-KG	NS	NS	*(13.1%)
	Succinic acid*	NS	NS	NS	Succinic acid*	NS	NS	NS
	Fumaric acid	*(9.64%)	NS	NS	Fumaric acid	*** (21.4%)	NS	NS
	Malic acid	*(7.22%)	*(11.6%)	** (15.6%)	Malic acid	**** (26.7%)	NS	NS
Urea cycle	Urea*	NS	NS	NS	Urea	*(9.95%)	** (19.4%)	NS
	Ornithine	**** (24%)	NS	NS	Ornithine	**** (27.2%)	NS	NS
	Putrescine*	NS	NS	NS	Putrescine	*** (24.8%)	NS	NS
	Citrulline	**** (27.7%)	NS	*(8.81%)	Citrulline	*** (21.2%)	NS	NS
	N-Acetylglutamate	*** (18.7%)	NS	** (14.8%)	N-Acetylglutamate*	NS	NS	NS
Amino acids	Alanine	*** (21.5%)	NS	NS	Alanine*	NS	NS	NS
	Aspartic acid	**** (77.4%)	NS	NS	Aspartic acid	NS	NS	** (20.7%)
	Asparagine	*(10%)	NS	NS	Asparagine*	NS	NS	NS
	Cysteine	NS	** (23.5%)	*(9.77%)	Cysteine	N/A	N/A	N/A
	Cystine	**** (62.7%)	NS	*(5.98%)	Cystine	*(6.58%)	NS	NS

... continued

Amino acids	Glutamic acid	**** (79.5%)	*(2.34%)	*(2.23%)	Glutamic acid	NS	NS	*(11.2%)
	Glutamine	**** (60%)	NS	NS	Glutamine	*** (15.9%)	NS	*** (27%)
	Glycine	NS	NS	*(11.7%)	Glycine*	NS	NS	NS
	Histidine	NS	NS	*(12.5%)	Histidine	*** (20.2%)	*(10.5%)	*(8.47%)
	Isoleucine*	NS	NS	NS	Isoleucine	NS	NS	*** (28.8%)
	Leucine	*(10.7%)	NS	NS	Leucine	NS	NS	** (21.3%)
	Lysine	NS	NS	*(14.6%)	Lysine	*** (24.3%)	NS	NS
	Methionine	**** (68%)	** (6.22%)	NS	Methionine	*** (20.4%)	NS	NS
	Phenylalanine	*(11.8%)	NS	NS	Phenylalanine	*(8.02%)	NS	*(15.8%)
	Proline	**** (39.7%)	** (10.7%)	NS	Proline	NS	NS	*(11.7%)
	Serine	NS	*(12.6%)	*(14.1%)	Serine	NS	NS	** (20.6%)
	Threonine	*** (20.4%)	NS	** (16.2%)	Threonine*	NS	NS	NS
	Tyrosine	** (17.9%)	NS	*(14.2%)	Tyrosine	** (15.3%)	NS	NS
	Tryptophan	NS	NS	*** (26.2%)	Tryptophan	*** (22.4%)	NS	** (14.8%)
	Valine	*(7.22%)	NS	NS	Valine	NS	NS	** (17.8%)
Fatty acids, cholesterol & glycerol	2-Ketoisocaproic acid	** (17.2%)	NS	NS	2-Ketoisocaproic acid	*(10.4%)	NS	NS
	Cholesterol	**** (52.3%)	NS	NS	Cholesterol	** (12.3%)	NS	** (20.7%)
	Docosahexaenoic acid	**** (65.5%)	NS	NS	Docosahexaenoic acid*	NS	NS	NS
	Arachidonic acid	**** (63.4%)	NS	NS	Arachidonic acid	** (17.3%)	NS	NS
	Heptadecanoic acid	** (12.7%)	NS	NS	Heptadecanoic acid	NS	NS	** (22.6%)
	Stearic acid	**** (48.2%)	NS	NS	Stearic acid*	NS	NS	NS
	Myristic acid	**** (48.6%)	*(5.71%)	*(5.95%)	Myristic acid	** (15.6%)	NS	** (15.9%)
	Palmitic acid	**** (54.4%)	*(5.64%)	NS	Palmitic acid	**** (38%)	NS	NS
	Palmitoleic acid	**** (51.4%)	** (10.5%)	*(6.24%)	Palmitoleic acid*	NS	NS	NS
	Oleic acid	**** (45.2%)	*(7.18%)	*(6.65%)	Oleic acid	*(6.8%)	NS	NS
	Linoleic acid	**** (48.3%)	NS	NS	Linoleic acid	*(8.51%)	NS	NS
	Linolenic acid	** (19.1%)	NS	NS	Linolenic acid	NS	** (22.5%)	NS
	Pantothenic acid	**** (34.8%)	NS	NS	Pantothenic acid	NS	NS	*(12.8%)
	Glycerol	**** (59%)	**** (13%)	NS	Glycerol	** (18.7%)	NS	*(10.7%)
	3-Hydroxybutyrate	*** (20.8%)	NS	NS	3-Hydroxybutyrate*	NS	NS	NS
Reodx & Others	4-Hydroxybutyrate	*** (19.1%)	NS	NS	4-Hydroxybutyrate	*(12.3%)	NS	NS
	Nicotinamide	*(17.1%)	NS	NS	Nicotinamide	NS	NS	*(11.1%)
	Glutathione	** (14.3%)	NS	NS	Glutathione	*(7.45%)	NS	NS
	Taurine	**** (43.4%)	NS	NS	Taurine	*** (21%)	NS	NS
	Methionine sulfox.*	NS	NS	NS	Methionine sulfox.	**** (26.6%)	*(9.42%)	NS
	AMP*	NS	NS	NS	AMP	*** (19.5%)	NS	** (17%)

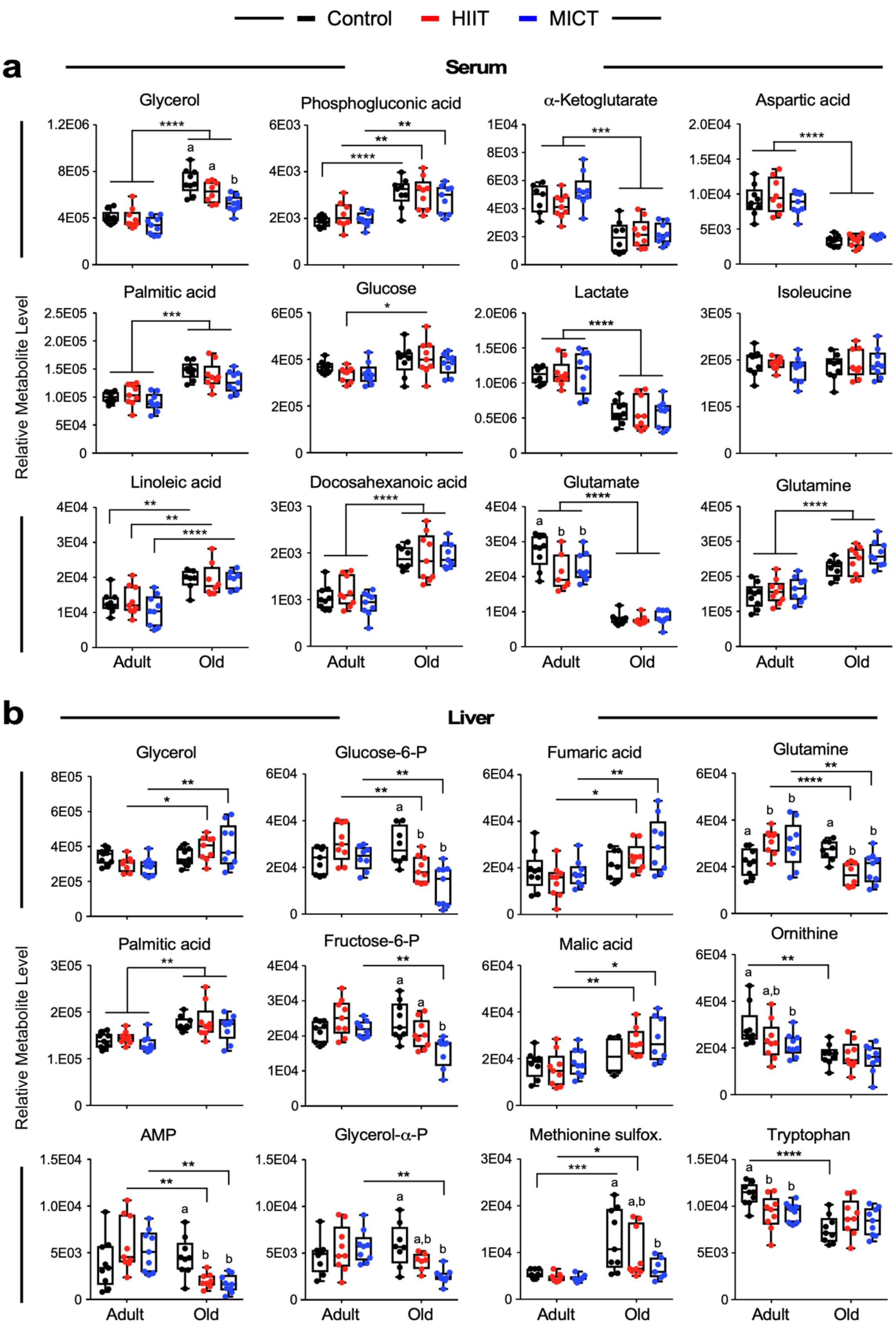
Control
HIIT
MICT
a
Serum
Glycerol
Phosphogluconic acid
α-Ketoglutarate
Aspartic acid
Palmitic acid
Glucose
Lactate
Isoleucine
Linoleic acid
Docosahexanoic acid
Glutamate
Glutamine
Relative Metabolite Level
Adult
Old
b
Liver
Glycerol
Glucose-6-P
Fumaric acid
Glutamine
Palmitic acid
Fructose-6-P
Malic acid
Ornithine
AMP
Glycerol-α-P
Methionine sulfox.
Tryptophan

Supplementary Figure 9. Profile of select metabolites in serum and liver from sedentary (CON) and exercised adult and old mice. Relative level of the indicated metabolites in serum (**a**) and liver (**b**) is depicted as box and whisker plots (n = 9 per group), with minimum, lower quartile (Q1), median (Q2), upper quartile (Q3), and maximum values. Adult, 5 months of age; Old, 24 months of age. CON, black symbols; HIIT, red symbols; MICT, blue symbols. * $p \leq 0.05$, ** $p \leq 0.01$, *** $p \leq 0.001$, **** $p \leq 0.0001$ by two-way ANOVA with the Sidak post-hoc test. Two variables with different lowercase letters have a statistically significant relationship with $p \leq 0.05$. Related to Fig. 5 and 6.

SUPPLEMENTARY TABLES

Supplementary Table 1. Impact of age and exercise on metabolic parameters

	**Adult (n= 5)**		
	AUC-RER	AUC-EE	AUC-Activity
CON	20.93 ± 0.3115	28.97 ± 0.4228	35966 ± 2192
HIIT	19.43 ± 0.305[a]	23.45 ± 0.3323[a]	31993 ± 1691
MICT	18.79 ± 0.2837[a]	25.95 ± 0.7516[a,b]	36545 ± 2157
One-way ANOVA	*P* = 0.0009	*P* < 0.0001	*P* = 0.1862
	**Old (n = 9)**		
	AUC-RER	AUC-EE	AUC-Activity
CON	7.854 ± 0.2064	9.617 ± 0.277	31920 ± 2310
HIIT	7.499 ± 0.2174	8.778 ± 0.2475	21655 ± 1836
MICT	8.045 ± 0.1816[b]	7.664 ± 0.3027[a,b]	47443 ± 3930[a,b]
One-way ANOVA	*P* = 0.0381	*P* = 0.0003	*P* < 0.0001

Areas under the curve (AUC) were calculated from the averaged, hourly trajectories of the indicated parameters (RER, EE, and locomotion) captured during two light/dark cycles in adult and old mice. Values are expressed as mean ± SEM. Data were analyzed by one-way ANOVA coupled with Tukey's post-hoc test. [a]p < 0.05 vs. CON; [b]p < 0.05 vs. HIIT. Related to Figure 1d.

Supplementary Table 2. Regression-based analysis of Energy Expenditure (ANCOVA).

**ADULT**	Light phase		Dark phase	
	Equation	(DFn,DFd), p	Equation	(DFn,DFd), p
CON	Y=1.776e-005*X+0.5086	(1,3); p=0.9977	Y=-2.115e-005*X+0.5817	(1,3); p=0.9971
HIIT	Y=-0.003437*X+0.5410	(1,3); p=0.6896	Y=0.001233*X+0.4853	(1,3); p=0.9048
MICT	Y=0.01251*X+0.08830	(1,3); p=0.784	Y=0.01706*X+0.02264	(1,3); p=0.698
**OLD**	Light phase		Dark phase	
	Equation	(DFn,DFd), p	Equation	(DFn,DFd), p
CON	Y=0.009512*X+0.2113	(1,7); p=0.0104	Y=0.006955*X+0.3807	(1,7); p=0.0236
HIIT	Y=0.003681*X+0.3630	(1,7); p=0.081	Y=0.0004281*X+0.5584	(1,7); p=0.8888
MICT	Y=0.001417*X+0.03947	(1,7); p=1127	Y=01529*X+0.09013	(1,7); p=0.2091

Related to Figure 1h and Supplementary Figure 1f.

Supplementary Table 3. Effect of age (Adult vs. Old), exercise regimen (CON, HIIT, MICT), and interaction 'age x exercise' on OXPHOS activity *in vitro*.

	OXPHOS activity		
Effect	Age	Exercise	Interaction
C-I	p<0.0001****	P=0.0003***	P=0.2044
C-II	P=0.0069**	p<0.0001****	P=0.8856
C-III	P=0.8617	P=0.0001***	P=0.0006***
C-IV	p<0.0001****	P=0.4439	P=0.0504
C-V	P=0.0092**	P=0.6132	P=0.0524

Note: The results were analyzed by two-way ANOVA with the Sidak post-hoc test. N =5 per group. Related to Figure 3d.

Supplementary Table 4. List of GO terms (category: biological processes) shared by the skeletal muscle of adult and old mice in response to a 4-week exercise training vs. sedentary controls (CON).

BIOLOGICAL PROCESSES		HIIT-CON					
		Adult			OLD		
Gene Ontology Term	No. Genes	Zscore	(P_value)	(fdr)	Zscore	(P_value)	(fdr)
GO0031424 KERATINIZATION	40	**18.0724**	1.14E-08	1.45E-06	**7.0065**	0.00013	0.00359
GO0008544 EPIDERMIS DEVELOPMENT	59	**8.4313**	0.00031	0.00892	**2.9852**	0.03457	0.22261
GO0002009 MORPHOGENESIS OF AN EPITHELIUM	16	**4.8488**	0.03477	0.24908	**3.5102**	0.00180	0.02853
GO0000038 VERY LONG CHAIN FATTY ACID METABOLIC PRO	9	**2.5985**	0.00643	0.08001	**4.5534**	0.01414	0.12755
GO0006099 TRICARBOXYLIC ACID CYCLE	24	**2.4604**	5.01E-08	4.74E-06	**1.6292**	0.00011	0.00318
GO0009416 RESPONSE TO LIGHT STIMULUS	5	**2.3749**	0.00079	0.01774	**2.7843**	0.03198	0.21172
GO0007155 CELL ADHESION	507	**4.9571**	9.21E-06	0.00046	**-3.0828**	0.00645	0.07790
GO0006954 INFLAMMATORY RESPONSE	191	**4.1943**	4.14E-05	0.00178	**-7.2674**	7.15E-08	4.30E-06
GO0008152 METABOLIC PROCESS	524	**4.1889**	9.17E-05	0.00326	**-2.0011**	0.02957	0.20266
GO0030049 MUSCLE FILAMENT SLIDING	6	**3.9752**	4.21E-05	0.00179	**-1.8838**	0.04939	0.27382
GO0006508 PROTEOLYSIS	492	**3.3357**	0.00164	0.03025	**-3.0586**	0.00538	0.06770
GO0007165 SIGNAL TRANSDUCTION	2259	**3.0138**	0.00244	0.04164	**-6.1007**	1.13E-07	6.44E-06
GO0007204 ELEVATION OF CYTOSOLIC CALCIUM ION CONCE	38	**2.8960**	0.00127	0.02496	**-3.9930**	0.00693	0.08161
GO0032781 POSITIVE REGULATION OF ATPASE ACTIVITY	3	**2.7039**	9.08E-06	0.00046	**-3.4311**	0.00259	0.03813
GO0045059 POSITIVE THYMIC T CELL SELECTION	8	**2.6310**	2.85E-05	0.00126	**-4.0346**	0.00051	0.01051
GO0007186 G PROTEIN COUPLED RECEPTOR PROTEIN SIGNA	1582	**2.6142**	0.00977	0.10811	**-3.8871**	0.00093	0.01730
GO0006952 DEFENSE RESPONSE	171	**2.5901**	0.02075	0.17841	**-7.7999**	8.94E-09	6.17E-07
GO0007166 CELL SURFACE RECEPTOR LINKED SIGNAL TRAN	164	**2.5236**	0.00786	0.09261	**-7.2324**	1.02E-09	8.46E-08
GO0007242 INTRACELLULAR SIGNALING CASCADE	398	**2.4840**	0.00184	0.03275	**-4.5903**	9.52E-06	0.00036
GO0008015 CIRCULATION	36	**2.4793**	0.00177	0.03176	**-2.4045**	0.04247	0.25329
GO0008283 CELL PROLIFERATION	257	**2.4665**	0.01035	0.11159	**-4.2423**	2.57E-05	0.00089
GO0048535 LYMPH NODE DEVELOPMENT	22	**2.3843**	0.04851	0.28983	**-5.4531**	0.00309	0.04413
GO0006693 PROSTAGLANDIN METABOLIC PROCESS	12	**2.2236**	0.00982	0.10835	**-3.2462**	0.00052	0.01058
GO0007599 HEMOSTASIS	10	**2.1709**	0.03078	0.22949	**-2.8166**	0.00733	0.08478
GO0006928 CELL MOTILITY	95	**1.8016**	0.03795	0.26064	**-4.4872**	7.06E-05	0.00211
GO0043123 POSITIVE REGULATION OF I KAPPAB KINASE O	61	**1.6975**	0.01722	0.15785	**-3.1457**	0.00174	0.02767
GO0006366 TRANSCRIPTION FROM RNA POLYMERASE II PRO	158	**-2.0111**	0.01151	0.11982	**2.1051**	0.02755	0.19527
GO0006355 REGULATION OF TRANSCRIPTION DNA DEPENDE	1706	**-2.9134**	0.00060	0.01478	**2.6094**	0.00384	0.05168
GO0006350 TRANSCRIPTION	1379	**-3.6244**	6.26E-06	0.00035	**2.2881**	0.00843	0.09244

		MICT-CON					
		Adult			OLD		
	No. Genes	Zscore	(P_value)	(fdr)	Zscore	(P_value)	(fdr)
GO0007608 SENSORY PERCEPTION OF SMELL	1084	**25.0377**	1.42E-190	2.35E-187	**8.4377**	1.21E-29	2.37E-27
GO0007186 G PROTEIN COUPLED RECEPTOR PROTEIN SIGNA	1582	**24.6426**	1.53E-162	1.01E-159	**3.3216**	0.00094	0.00954
GO0050896 RESPONSE TO STIMULUS	261	**7.8904**	2.51E-20	9.89E-19	**3.2814**	0.00021	0.00280
GO0007268 SYNAPTIC TRANSMISSION	144	**2.3087**	0.01878	0.05826	**2.1013**	0.00841	0.05763
GO0006817 PHOSPHATE TRANSPORT	70	**-1.9543**	0.01113	0.03807	**-4.6252**	0.00460	0.03519
GO0007160 CELL MATRIX ADHESION	65	**-2.0832**	0.01763	0.05549	**-5.2730**	7.43E-05	0.00116
GO0006633 FATTY ACID BIOSYNTHETIC PROCESS	42	**-2.1336**	0.01578	0.05084	**-3.7503**	0.02511	0.13008
GO0007275 MULTICELLULAR ORGANISMAL DEVELOPMENT	901	**-2.2158**	0.01130	0.03850	**-2.9722**	0.00530	0.03954
GO0051258 PROTEIN POLYMERIZATION	18	**-2.3097**	0.01792	0.05616	**-3.4755**	0.00271	0.02346
GO0008610 LIPID BIOSYNTHETIC PROCESS	81	**-2.3572**	0.02291	0.06913	**-5.0088**	0.00021	0.00280
GO0051272 POSITIVE REGULATION OF CELL MOTILITY	5	**-2.3601**	3.55E-05	0.00024	**-2.0941**	0.00621	0.04456
GO0007017 MICROTUBULE BASED PROCESS	59	**-2.4729**	0.00685	0.02514	**-3.0149**	0.00858	0.05830
GO0007018 MICROTUBULE BASED MOVEMENT	72	**-2.4779**	0.01254	0.04180	**-4.1889**	0.00065	0.00706
GO0006801 SUPEROXIDE METABOLIC PROCESS	14	**-2.6088**	7.21E-05	0.00046	**-3.6499**	0.00041	0.00492
GO0030049 MUSCLE FILAMENT SLIDING	6	**-2.6354**	0.01160	0.03933	**-4.1153**	8.06E-09	2.93E-07
GO0006917 INDUCTION OF APOPTOSIS	99	**-2.6822**	0.00069	0.00350	**-3.0746**	0.01746	0.10018
GO0008360 REGULATION OF CELL SHAPE	61	**-2.8094**	0.00526	0.02018	**-2.9822**	0.00411	0.03243
GO0000910 CYTOKINESIS	40	**-3.0686**	2.22E-05	0.00016	**-4.5943**	0.00327	0.02689
GO0008283 CELL PROLIFERATION	257	**-3.7332**	3.72E-05	0.00025	**-5.0007**	1.37E-05	0.00026
GO0030036 ACTIN CYTOSKELETON ORGANIZATION AND BIOG	106	**-3.9289**	1.86E-06	1.68E-05	**-2.6661**	0.00940	0.06276
GO0043123 POSITIVE REGULATION OF I KAPPAB KINASE O	61	**-4.0414**	7.05E-10	1.09E-08	**-2.4085**	0.04453	0.19653
GO0000074 REGULATION OF PROGRESSION THROUGH CELL C	259	**-4.1482**	2.03E-05	0.00014	**-4.6998**	0.00054	0.00617
GO0006461 PROTEIN COMPLEX ASSEMBLY	112	**-4.1543**	5.30E-07	5.41E-06	**-4.4474**	0.00196	0.01785
GO0007010 CYTOSKELETON ORGANIZATION AND BIOGENESIS	119	**-4.3243**	5.50E-06	4.38E-05	**-4.5968**	0.00366	0.02958
GO0006928 CELL MOTILITY	95	**-4.4486**	1.19E-06	1.13E-05	**-5.3705**	0.00046	0.00536
GO0007067 MITOSIS	146	**-4.5622**	5.63E-09	7.66E-08	**-8.5441**	4.76E-08	1.50E-06
GO0007242 INTRACELLULAR SIGNALING CASCADE	398	**-4.7651**	1.16E-07	1.30E-06	**-5.6567**	1.01E-05	0.00020
GO0051301 CELL DIVISION	206	**-5.0210**	2.58E-10	4.25E-09	**-7.5704**	1.34E-07	3.83E-06
GO0042981 REGULATION OF APOPTOSIS	126	**-5.1856**	3.95E-15	1.07E-13	**-2.8722**	0.01399	0.08526
GO0006468 PROTEIN AMINO ACID PHOSPHORYLATION	555	**-6.1124**	1.84E-12	3.91E-11	**-3.3688**	0.00263	0.02295
GO0008152 METABOLIC PROCESS	524	**-6.9670**	1.01E-13	2.40E-12	**-2.6134**	0.01234	0.07723
GO0007049 CELL CYCLE	443	**-7.4224**	1.36E-19	5.17E-18	**-5.1284**	5.53E-05	0.00092
GO0006915 APOPTOSIS	406	**-8.2222**	1.75E-21	7.16E-20	**-4.9040**	0.00020	0.00273
GO0042742 DEFENSE RESPONSE TO BACTERIUM	69	**3.2911**	0.00146	0.00672	**-4.6060**	0.00283	0.02420
GO0006952 DEFENSE RESPONSE	171	**2.7677**	0.01200	0.04040	**-11.6769**	1.38E-12	7.27E-11
GO0042130 NEGATIVE REGULATION OF T CELL PROLIFERAT	18	**2.6999**	0.00315	0.01315	**-3.4470**	0.03871	0.17748
GO0031424 KERATINIZATION	40	**2.4504**	0.00046	0.00241	**-11.4263**	3.22E-06	6.96E-05
GO0007204 ELEVATION OF CYTOSOLIC CALCIUM ION CONCE	38	**2.2531**	0.02827	0.08207	**-5.8836**	0.00158	0.01491
GO0030101 NATURAL KILLER CELL ACTIVATION	10	**2.1832**	0.00014	0.00083	**-3.8412**	0.04710	0.20513
GO0006777 MO MOLYBDOPTERIN COFACTOR BIOSYNTHETIC P	7	**-2.2261**	1.67E-06	1.51E-05	**2.5746**	0.00090	0.00918
GO0048511 RHYTHMIC PROCESS	25	**-2.6943**	1.09E-05	8.10E-05	**3.0043**	0.00424	0.03335

GO0008033 TRNA PROCESSING	49	**-2.8728**	0.00012	0.00076	**2.6036**	3.70E-07	9.42E-06
GO0006417 REGULATION OF TRANSLATION	49	**-3.1207**	1.52E-05	0.00011	**2.3383**	0.00222	0.01978
GO0008286 INSULIN RECEPTOR SIGNALING PATHWAY	33	**-3.2265**	1.11E-05	8.19E-05	**2.2215**	0.01282	0.07976
GO0006366 TRANSCRIPTION FROM RNA POLYMERASE II PRO	158	**-3.2488**	0.00023	0.00132	**3.9705**	0.00020	0.00265
GO0006446 REGULATION OF TRANSLATIONAL INITIATION	19	**-3.7599**	7.36E-06	5.58E-05	**2.4797**	1.48E-07	4.12E-06
GO0006418 TRNA AMINOACYLATION FOR PROTEIN TRANSLAT	49	**-4.3518**	7.38E-09	9.81E-08	**2.5453**	0.00165	0.01547
GO0006396 RNA PROCESSING	70	**-4.8048**	9.99E-11	1.77E-09	**3.0492**	2.59E-07	6.80E-06
GO0006413 TRANSLATIONAL INITIATION	22	**-4.8982**	2.89E-18	9.77E-17	**2.0082**	8.54E-14	5.23E-12
GO0006364 RRNA PROCESSING	55	**-4.9529**	4.57E-09	6.33E-08	**2.0947**	0.00660	0.04688
GO0006631 FATTY ACID METABOLIC PROCESS	71	**-5.2885**	2.45E-08	3.02E-07	**2.1069**	0.04763	0.20555
GO0016568 CHROMATIN MODIFICATION	123	**-5.5605**	1.09E-14	2.85E-13	**2.1414**	0.00272	0.02354
GO0006511 UBIQUITIN DEPENDENT PROTEIN CATABOLIC PR	116	**-6.5071**	1.39E-17	4.61E-16	**3.4183**	6.71E-05	0.00107
GO0006099 TRICARBOXYLIC ACID CYCLE	24	**-6.5886**	9.77E-11	1.74E-09	**2.2095**	1.63E-12	8.42E-11
GO0006888 ER TO GOLGI VESICLE MEDIATED TRANSPORT	74	**-6.8861**	1.43E-29	8.31E-28	**2.3009**	0.00017	0.00237
GO0006355 REGULATION OF TRANSCRIPTION DNA DEPENDE	1706	**-7.6911**	4.89E-16	1.51E-14	**2.9322**	0.00289	0.02449
GO0006464 PROTEIN MODIFICATION PROCESS	192	**-8.0830**	6.58E-25	3.07E-23	**2.3090**	0.00896	0.06025
GO0006350 TRANSCRIPTION	1379	**-8.6125**	1.45E-20	5.86E-19	**3.2763**	0.00081	0.00843
GO0006397 MRNA PROCESSING	197	**-8.6354**	2.16E-31	1.38E-29	**4.5793**	5.94E-20	6.34E-18
GO0008380 RNA SPLICING	160	**-8.7525**	3.05E-35	2.24E-33	**4.3724**	4.36E-22	4.98E-20
GO0042254 RIBOSOME BIOGENESIS AND ASSEMBLY	86	**-8.8087**	5.39E-16	1.65E-14	**2.8511**	1.20E-08	4.27E-07
GO0006512 UBIQUITIN CYCLE	365	**-11.1826**	3.25E-51	3.98E-49	**4.4941**	2.24E-07	5.92E-06
GO0015031 PROTEIN TRANSPORT	462	**-12.1593**	3.34E-50	3.95E-48	**2.8125**	0.00067	0.00726
GO0006412 TRANSLATION	268	**-16.6741**	1.35E-83	2.78E-81	**4.2605**	1.24E-15	1.00E-13

Significance defined as Zscore > 1.5 in either direction, p value ≤ 0.05, and false discovery rate (fdr) with cut-off value of 0.3. Abbreviations: CON, sedentary controls; HIIT, high-intensity interval training; MICT, moderate-intensity continuous training. Related to Figure 4b, c.

Supplementary Table 5a. List of top biological processes (GO terms) that are either shared or unique in skeletal muscle of sedentary (CON) mice and mice on MICT in the (Old-Adult) pairwise comparison.

	# Genes	(Old-Young)_CON			(Old-Young)_MICT		
(1)Gene Ontology Term	in GOTerm	Zscore	(P_value)	(fdr)	Zscore	(P_value)	(fdr)
GO0006915 APOPTOSIS	406	**5.9937**	2.04E-06	6.04E-05	**8.4234**	7.34E-22	3.38E-20
GO0008152 METABOLIC PROCESS	524	**2.5152**	0.0176	0.1041	**6.6933**	9.76E-13	2.29E-11
GO0007049 CELL CYCLE	443	**3.7206**	0.0004	0.0061	**6.4760**	1.46E-13	3.71E-12
GO0042981 REGULATION OF APOPTOSIS	126	**3.4496**	0.0026	0.0249	**5.2667**	1.63E-14	4.56E-13
GO0006928 CELL MOTILITY	95	**6.1462**	0.0001	0.0020	**4.5856**	2.65E-07	3.40E-06
GO0006916 ANTI APOPTOSIS	122	**2.8099**	0.0042	0.0358	**4.4039**	2.12E-05	0.0002
GO0043123 POSITIVE REGULATION OF I KAPPAB KINASE O	61	**3.3362**	0.0019	0.0192	**4.3232**	2.47E-10	4.72E-09
GO0007242 INTRACELLULAR SIGNALING CASCADE	398	**5.1768**	1.31E-05	0.0003	**4.3046**	3.17E-06	3.35E-05
GO0006629 LIPID METABOLIC PROCESS	226	**4.7033**	0.0002	0.0027	**4.2327**	4.45E-05	0.0004
GO0043065 POSITIVE REGULATION OF APOPTOSIS	59	**2.7238**	0.0164	0.0993	**3.7992**	1.38E-07	1.88E-06
GO0051301 CELL DIVISION	206	**5.4397**	5.45E-06	0.0002	**3.7456**	1.81E-05	0.0002
GO0006461 PROTEIN COMPLEX ASSEMBLY	112	**3.7333**	0.0097	0.0670	**3.6267**	1.41E-05	0.0001
GO0000074 REGULATION OF PROGRESSION THROUGH CELL C	259	**3.9244**	0.0007	0.0093	**3.5847**	0.0005	0.0029
GO0007067 MITOSIS	146	**6.5977**	1.00E-06	3.14E-05	**3.3636**	0.0002	0.0011
GO0008283 CELL PROLIFERATION	257	**4.3608**	3.08E-05	0.0006	**3.2373**	0.0006	0.0035
GO0006917 INDUCTION OF APOPTOSIS	99	**3.5998**	0.0056	0.0446	**2.7963**	0.0003	0.0019
GO0008610 LIPID BIOSYNTHETIC PROCESS	81	**5.4737**	0.0002	0.0027	**2.4094**	0.0187	0.0660
GO0006693 PROSTAGLANDIN METABOLIC PROCESS	12	**4.5228**	0.0014	0.0150	**2.3867**	0.0100	0.0396
GO0006801 SUPEROXIDE METABOLIC PROCESS	14	**3.0646**	0.0210	0.1162	**2.1951**	0.0019	0.0095
GO0006633 FATTY ACID BIOSYNTHETIC PROCESS	42	**4.0339**	0.0206	0.1151	**2.1333**	0.0164	0.0596
GO0051085 CHAPERONE COFACTOR DEPENDENT PROTEIN FOL	11	**3.8125**	0.0055	0.0442	**1.7547**	0.0216	0.0743
GO0008624 INDUCTION OF APOPTOSIS BY EXTRACELLULAR	21	**4.5471**	0.0013	0.0147	**1.7431**	0.0153	0.0565
GO0006412 TRANSLATION	268	**-1.8551**	0.0002	0.0027	**17.4724**	1.76E-83	3.64E-81
GO0015031 PROTEIN TRANSPORT	462	**-1.8238**	0.0184	0.1072	**12.3796**	8.88E-50	1.09E-47
GO0006512 UBIQUITIN CYCLE	365	**-3.2925**	2.92E-06	8.33E-05	**11.5603**	1.07E-46	1.18E-44
GO0006397 MRNA PROCESSING	197	**-2.9455**	2.45E-11	1.62E-09	**9.2800**	1.13E-34	8.54E-33
GO0008380 RNA SPLICING	160	**-3.1683**	4.45E-17	6.14E-15	**9.1900**	6.03E-37	4.87E-35
GO0006457 PROTEIN FOLDING	199	**-1.9414**	0.0206	0.1149	**8.8108**	8.03E-20	3.28E-18
GO0006350 TRANSCRIPTION	1379	**-4.0953**	9.06E-06	0.0002	**8.0878**	3.31E-17	1.18E-15
GO0006464 PROTEIN MODIFICATION PROCESS	192	**-2.0027**	0.0097	0.0673	**8.0747**	8.44E-22	3.83E-20
GO0006355 REGULATION OF TRANSCRIPTION DNA DEPENDE	1706	**-4.2175**	5.35E-06	0.0001	**6.9647**	9.02E-13	2.13E-11
GO0006888 ER TO GOLGI VESICLE MEDIATED TRANSPORT	74	**-1.9152**	3.20E-07	1.16E-05	**6.9445**	3.34E-27	2.05E-25
GO0016568 CHROMATIN MODIFICATION	123	**-2.3188**	0.0001	0.0022	**5.3868**	1.03E-12	2.39E-11
GO0000398 NUCLEAR MRNA SPLICING VIA SPLICEOSOME	39	**-1.6791**	1.44E-06	4.41E-05	**5.1639**	1.15E-18	4.53E-17
GO0042130 NEGATIVE REGULATION OF T CELL PROLIFERAT	18	**4.6153**	0.0047	0.0392	**-2.1622**	0.0226	0.0772
GO0006952 DEFENSE RESPONSE	171	**12.6903**	1.10E-14	1.10E-12	**-2.4741**	0.0239	0.0804
GO0007165 SIGNAL TRANSDUCTION	2259	**3.7174**	0.0006	0.0082	**-16.3428**	1.85E-62	2.67E-60
GO0007608 SENSORY PERCEPTION OF SMELL	1084	**-3.2035**	1.56E-05	0.0004	**-21.8054**	8.83E-147	3.25E-144
GO0006955 IMMUNE RESPONSE	351	**18.6035**	2.91E-31	8.02E-29	0	0.2203	0.4105
GO0031424 KERATINIZATION	40	**14.0853**	4.01E-06	0.0001	0	0.0335	0.1031
GO0007166 CELL SURFACE RECEPTOR LINKED SIGNAL TRAN	164	**11.0561**	3.46E-10	2.12E-08	0	0.1213	0.2712
GO0006954 INFLAMMATORY RESPONSE	191	**10.5538**	1.65E-12	1.36E-10	0	0.2987	0.5075
GO0006968 CELLULAR DEFENSE RESPONSE	49	**10.3324**	8.17E-08	3.22E-06	0	0.5656	0.7531
GO0042113 B CELL ACTIVATION	25	**9.2327**	7.67E-05	0.0014	0	0.1311	0.2856
GO0006935 CHEMOTAXIS	95	**9.0868**	4.94E-08	2.02E-06	0	0.6750	0.8263
GO0042110 T CELL ACTIVATION	27	**8.5442**	4.53E-05	0.0009	0	0.2566	0.4584
GO0019882 ANTIGEN PROCESSING AND PRESENTATION	32	**8.4833**	2.18E-08	9.48E-07	0	0.1591	0.3287
GO0019886 ANTIGEN PROCESSING AND PRESENTATION OF E	17	**8.4755**	3.26E-09	1.63E-07	0	0.5623	0.7515
GO0006959 HUMORAL IMMUNE RESPONSE	30	**7.9932**	2.27E-05	0.0005	0	0.5605	0.7502
GO0048535 LYMPH NODE DEVELOPMENT	22	**7.6531**	0.0025	0.0248	0	0.8968	0.9471
GO0050853 B CELL RECEPTOR SIGNALING PATHWAY	9	**7.4290**	0.0002	0.0032	0	0.2674	0.4721
GO0008544 EPIDERMIS DEVELOPMENT	59	**7.3472**	0.0007	0.0088	0	0.6958	0.8387
GO0045087 INNATE IMMUNE RESPONSE	71	**7.3416**	5.55E-07	1.89E-05	0	0.1061	0.2445
GO0031295 T CELL COSTIMULATION	9	**7.2887**	9.27E-06	0.0002	0	0.1364	0.2941
GO0007155 CELL ADHESION	507	**7.1224**	2.58E-07	9.61E-06	0	0.1698	0.3429
GO0045059 POSITIVE THYMIC T CELL SELECTION	8	**6.9280**	0.0014	0.0151	0	0.8447	0.9237
GO0002504 ANTIGEN PROCESSING AND PRESENTATION OF P	8	**6.7837**	7.95E-15	8.22E-13	0	0.5705	0.7553
GO0006691 LEUKOTRIENE METABOLIC PROCESS	12	**6.3417**	0.0044	0.0368	0	0.4545	0.6561
GO0030216 KERATINOCYTE DIFFERENTIATION	36	**6.2518**	0.0059	0.0465	0	0.8600	0.9312
GO0042535 POSITIVE REGULATION OF TUMOR NECROSIS FA	8	**6.1024**	0.0005	0.0074	0	0.5766	0.7595
GO0001879 DETECTION OF YEAST	3	**6.0474**	2.51E-31	7.56E-29	0	0.0026	0.0123
GO0019370 LEUKOTRIENE BIOSYNTHETIC PROCESS	19	**5.9306**	0.0007	0.0086	0	0.1073	0.2465
GO0050776 REGULATION OF IMMUNE RESPONSE	25	**5.8333**	0.0004	0.0054	0	0.7758	0.8855

[illegible]	[illegible]	[illegible]	[illegible]	[illegible]	[illegible]	[illegible]	[illegible]
GO0042254 RIBOSOME BIOGENESIS AND ASSEMBLY	86	0	0.0023	0.0227	**9.3253**	1.30E-17	4.78E-16
GO0006810 TRANSPORT	1485	0	0.1403	0.3883	**9.3205**	9.82E-20	3.96E-18
GO0006886 INTRACELLULAR PROTEIN TRANSPORT	224	0	0.7458	0.8763	**9.1403**	1.44E-25	8.07E-24
GO0006511 UBIQUITIN DEPENDENT PROTEIN CATABOLIC PR	116	0	0.0270	0.1404	**7.3329**	5.44E-21	2.31E-19
GO0008150 BIOLOGICAL PROCESS	1466	0	0.9187	0.9638	**7.2553**	1.60E-08	2.50E-07
GO0006099 TRICARBOXYLIC ACID CYCLE	24	0	0.0016	0.0172	**6.8510**	2.30E-11	4.87E-10
GO0016192 VESICLE MEDIATED TRANSPORT	140	0	0.6579	0.8321	**6.4225**	1.17E-14	3.46E-13
GO0006118 ELECTRON TRANSPORT	367	0	0.1360	0.3817	**6.3544**	9.43E-10	1.70E-08
GO0006631 FATTY ACID METABOLIC PROCESS	71	0	0.9843	0.9924	**6.1938**	1.61E-10	3.13E-09
GO0015992 PROTON TRANSPORT	56	0	0.9816	0.9915	**5.9916**	3.71E-08	5.46E-07
GO0006950 RESPONSE TO STRESS	111	0	0.0590	0.2316	**5.9046**	7.19E-08	1.02E-06
GO0015986 ATP SYNTHESIS COUPLED PROTON TRANSPORT	33	0	0.5760	0.7849	**5.8887**	5.43E-07	6.53E-06
GO0006974 RESPONSE TO DNA DAMAGE STIMULUS	170	0	0.6774	0.8413	**5.8580**	7.23E-14	1.91E-12
GO0006396 RNA PROCESSING	70	0	0.0026	0.0249	**5.5222**	1.32E-12	3.02E-11
GO0006281 DNA REPAIR	189	0	0.9853	0.9928	**5.4789**	7.29E-11	1.44E-09
GO0006364 RRNA PROCESSING	55	0	0.0412	0.1842	**5.2711**	6.23E-09	1.04E-07
GO0006413 TRANSLATIONAL INITIATION	22	0	0.0003	0.0050	**5.2493**	6.48E-17	2.26E-15
GO0006468 PROTEIN AMINO ACID PHOSPHORYLATION	555	0	0.0726	0.2635	**5.1987**	8.36E-09	1.38E-07
GO0006936 MUSCLE CONTRACTION	71	0	0.5589	0.7727	**5.1611**	8.75E-07	1.03E-05
GO0006120 MITOCHONDRIAL ELECTRON TRANSPORT NADH T	6	0	0.0973	0.3138	**5.0815**	1.76E-223	1.46E-220
GO0006418 TRNA AMINOACYLATION FOR PROTEIN TRANSLAT	49	0	0.0747	0.2675	**4.9139**	1.23E-09	2.20E-08
GO0006754 ATP BIOSYNTHETIC PROCESS	28	0	0.9050	0.9570	**4.8632**	6.37E-05	0.0005
GO0065002 INTRACELLULAR PROTEIN TRANSPORT ACROSS A	63	0	0.0467	0.1988	**4.6911**	1.32E-09	2.35E-08
GO0005977 GLYCOGEN METABOLIC PROCESS	29	0	0.4924	0.7309	**4.6066**	5.28E-07	6.42E-06
GO0007517 MUSCLE DEVELOPMENT	77	0	0.6424	0.8233	**4.5939**	2.88E-06	3.08E-05
GO0015696 AMMONIUM TRANSPORT	3	0	0.5231	0.7515	**-6.1192**	1.04E-08	1.70E-07
GO0050896 RESPONSE TO STIMULUS	261	0	0.0619	0.2383	**-6.8769**	5.04E-15	1.58E-13
GO0007186 G PROTEIN COUPLED RECEPTOR PROTEIN SIGNA	1582	0	0.4112	0.6785	**-22.0310**	9.17E-136	3.04E-133

Supplementary Table 5b. List of top biological processes (GO terms) that are either shared or unique in skeletal muscle of sedentary (CON) mice and mice on HIIT in the (Old-Adult) pairwise comparison.

	# Genes	(Old-Young)_CON			(Old-Young)_HIIT		
(1)Gene Ontology Term	in GOTerm	Zscore	(P_value)	(fdr)	Zscore	(P_value)	(fdr)
GO0006955 IMMUNE RESPONSE	351	**18.6035**	2.91E-31	8.02E-29	**8.1916**	2.32E-10	2.20E-08
GO0031424 KERATINIZATION	40	**14.0853**	4.01E-06	0.0001	**10.2985**	0.0019	0.0315
GO0006952 DEFENSE RESPONSE	171	**12.6903**	1.10E-14	1.10E-12	**6.8315**	1.35E-05	0.0005
GO0007166 CELL SURFACE RECEPTOR LINKED SIGNAL TRAN	164	**11.0561**	3.46E-10	2.12E-08	**5.3151**	0.0002	0.0054
GO0006954 INFLAMMATORY RESPONSE	191	**10.5538**	1.65E-12	1.36E-10	**3.2793**	0.0118	0.1147
GO0006968 CELLULAR DEFENSE RESPONSE	49	**10.3324**	8.17E-08	3.22E-06	**5.9930**	0.0003	0.0069
GO0042113 B CELL ACTIVATION	25	**9.2327**	7.67E-05	0.0014	**3.8879**	0.0046	0.0581
GO0006935 CHEMOTAXIS	95	**9.0868**	4.94E-08	2.02E-06	**4.3039**	0.0058	0.0706
GO0042110 T CELL ACTIVATION	27	**8.5442**	4.53E-05	0.0009	**4.8957**	0.0039	0.0525
GO0019882 ANTIGEN PROCESSING AND PRESENTATION	32	**8.4833**	2.18E-08	9.48E-07	**2.6512**	0.0128	0.1210
GO0019886 ANTIGEN PROCESSING AND PRESENTATION OF E	17	**8.4755**	3.26E-09	1.63E-07	**3.0022**	0.0097	0.1025
GO0006959 HUMORAL IMMUNE RESPONSE	30	**7.9932**	2.27E-05	0.0005	**2.7066**	0.0406	0.2416
GO0050853 B CELL RECEPTOR SIGNALING PATHWAY	9	**7.4290**	0.0002	0.0032	**2.9740**	0.0324	0.2110
GO0008544 EPIDERMIS DEVELOPMENT	59	**7.3472**	0.0007	0.0088	**5.5360**	0.0070	0.0810
GO0045087 INNATE IMMUNE RESPONSE	71	**7.3416**	5.55E-07	1.89E-05	**2.7499**	0.0156	0.1366
GO0031295 T CELL COSTIMULATION	9	**7.2887**	9.27E-06	0.0002	**3.1815**	0.0105	0.1073
GO0002504 ANTIGEN PROCESSING AND PRESENTATION OF P	8	**6.7837**	7.95E-15	8.22E-13	**2.6624**	0.0007	0.0150
GO0007067 MITOSIS	146	**6.5977**	1.00E-06	3.14E-05	**2.4837**	0.0088	0.0946
GO0042535 POSITIVE REGULATION OF TUMOR NECROSIS FA	8	**6.1024**	0.0005	0.0074	**2.3560**	0.0452	0.2590
GO0001879 DETECTION OF YEAST	3	**6.0474**	2.51E-31	7.56E-29	**3.2001**	0.0020	0.0319
GO0006915 APOPTOSIS	406	**5.9937**	2.04E-06	6.04E-05	**2.8851**	0.0049	0.0612
GO0042742 DEFENSE RESPONSE TO BACTERIUM	69	**5.9235**	0.0002	0.0033	**4.0143**	0.0256	0.1870
GO0016064 IMMUNOGLOBULIN MEDIATED IMMUNE RESPONSE	16	**5.7196**	4.12E-07	1.45E-05	**1.9670**	0.0293	0.2029
GO0006910 PHAGOCYTOSIS RECOGNITION	7	**5.4625**	7.64E-05	0.0014	**2.2571**	0.0481	0.2671
GO0045060 NEGATIVE THYMIC T CELL SELECTION	10	**5.4425**	0.0003	0.0040	**2.1776**	0.0053	0.0656
GO0006744 UBIQUINONE BIOSYNTHETIC PROCESS	9	**-2.1067**	0.0003	0.0046	**-1.6376**	3.08E-11	3.29E-09
GO0007519 STRIATED MUSCLE DEVELOPMENT	37	**-2.5551**	7.61E-05	0.0014	**-2.4653**	0.0301	0.2064
GO0008380 RNA SPLICING	160	**-3.1683**	4.45E-17	6.14E-15	**-1.6090**	0.0002	0.0058
GO0007608 SENSORY PERCEPTION OF SMELL	1084	**-3.2035**	1.56E-05	0.0004	**-5.9903**	2.36E-12	3.55E-10

GO0048535 LYMPH NODE DEVELOPMENT	22	**7.6531**	0.0025	0.0248	0	0.2617	0.6108
GO0007155 CELL ADHESION	507	**7.1224**	2.58E-07	9.61E-06	0	0.0923	0.3867
GO0045059 POSITIVE THYMIC T CELL SELECTION	8	**6.9280**	0.0014	0.0151	0	0.1334	0.4572
GO0006691 LEUKOTRIENE METABOLIC PROCESS	12	**6.3417**	0.0044	0.0368	0	0.2470	0.5993
GO0030216 KERATINOCYTE DIFFERENTIATION	36	**6.2518**	0.0059	0.0465	0	0.0893	0.3804
GO0006928 CELL MOTILITY	95	**6.1462**	0.0001	0.0020	0	0.1260	0.4455
GO0019370 LEUKOTRIENE BIOSYNTHETIC PROCESS	19	**5.9306**	0.0007	0.0086	0	0.2965	0.6481
GO0050776 REGULATION OF IMMUNE RESPONSE	25	**5.8333**	0.0004	0.0054	0	0.2041	0.5543
GO0008610 LIPID BIOSYNTHETIC PROCESS	81	**5.4737**	0.0002	0.0027	0	0.3607	0.6945
GO0006508 PROTEOLYSIS	492	**5.3500**	3.31E-05	0.0007	0	0.2561	0.6065
GO0007242 INTRACELLULAR SIGNALING CASCADE	398	**5.1768**	1.31E-05	0.0003	0	0.7688	0.9121
GO0030225 MACROPHAGE DIFFERENTIATION	11	**5.1732**	0.0401	0.1817	0	0.2173	0.5709
GO0007204 ELEVATION OF CYTOSOLIC CALCIUM ION CONCE	38	**4.7734**	0.0151	0.0940	0	0.9991	0.9994
GO0045410 POSITIVE REGULATION OF INTERLEUKIN 6 BIO	7	**4.6684**	0.0070	0.0518	0	0.0771	0.3500
GO0008624 INDUCTION OF APOPTOSIS BY EXTRACELLULAR	21	**4.5471**	0.0013	0.0147	0	0.2157	0.5680
GO0015804 NEUTRAL AMINO ACID TRANSPORT	6	**4.5404**	0.0207	0.1153	0	0.7405	0.8981
GO0006693 PROSTAGLANDIN METABOLIC PROCESS	12	**4.5228**	0.0014	0.0150	0	0.1729	0.5101
GO0006958 COMPLEMENT ACTIVATION CLASSICAL PATHWAY	24	**4.4617**	0.0039	0.0340	0	0.0951	0.3919
GO0019221 CYTOKINE AND CHEMOKINE MEDIATED SIGNALIN	50	**4.4284**	0.0007	0.0093	0	0.4632	0.7549
GO0042100 B CELL PROLIFERATION	11	**4.3685**	0.0125	0.0812	0	0.1188	0.4303
GO0008283 CELL PROLIFERATION	257	**4.3608**	3.08E-05	0.0006	0	0.6136	0.8411
GO0006874 CELLULAR CALCIUM ION HOMEOSTASIS	62	**4.1538**	0.0022	0.0219	0	0.5277	0.7951
GO0007596 BLOOD COAGULATION	63	**4.1311**	0.0023	0.0229	0	0.3984	0.7171
GO0009617 RESPONSE TO BACTERIUM	9	**4.0743**	0.0190	0.1091	0	0.1731	0.5101
GO0006633 FATTY ACID BIOSYNTHETIC PROCESS	42	**4.0339**	0.0206	0.1151	0	0.5303	0.7947
GO0008033 TRNA PROCESSING	49	**-2.5986**	2.77E-07	1.02E-05	0	0.0086	0.0936
GO0006366 TRANSCRIPTION FROM RNA POLYMERASE II PRO	158	**-2.7418**	0.0031	0.0288	0	0.8919	0.9657
GO0006397 MRNA PROCESSING	197	**-2.9455**	2.45E-11	1.62E-09	0	0.0004	0.0081
GO0042220 RESPONSE TO COCAINE	4	**-2.9499**	9.52E-06	0.0003	0	0.8108	0.9315
GO0006512 UBIQUITIN CYCLE	365	**-3.2925**	2.92E-06	8.33E-05	0	0.2805	0.6291
GO0006350 TRANSCRIPTION	1379	**-4.0953**	9.06E-06	0.0002	0	0.8489	0.9445
GO0006355 REGULATION OF TRANSCRIPTION DNA DEPENDE	1706	**-4.2175**	5.35E-06	0.0001	0	0.5125	0.7838
GO0000038 VERY LONG CHAIN FATTY ACID METABOLIC PRO	9	0	0.2262	0.4988	**4.4916**	0.0047	0.0590
GO0009247 GLYCOLIPID BIOSYNTHETIC PROCESS	5	0	0.2315	0.5058	**4.1699**	0.0133	0.1241
GO0001676 LONG CHAIN FATTY ACID METABOLIC PROCESS	12	0	0.2155	0.4883	**4.1139**	0.0105	0.1077
GO0015807 L AMINO ACID TRANSPORT	7	0	0.4855	0.7262	**4.0445**	0.0086	0.0936
GO0007010 CYTOSKELETON ORGANIZATION AND BIOGENESIS	119	0	0.4480	0.7035	**3.3317**	0.0255	0.1870
GO0006308 DNA CATABOLIC PROCESS	5	0	0.5867	0.7907	**3.3003**	0.0002	0.0059
GO0030101 NATURAL KILLER CELL ACTIVATION	10	0	0.0685	0.2551	**2.8708**	0.0102	0.1061
GO0006099 TRICARBOXYLIC ACID CYCLE	24	0	0.0016	0.0172	**-2.2370**	2.86E-08	1.75E-06
GO0007507 HEART DEVELOPMENT	145	0	0.0568	0.2252	**-2.4042**	0.0043	0.0559
GO0048661 POSITIVE REGULATION OF SMOOTH MUSCLE CEL	6	0	0.4364	0.6985	**-2.4122**	0.0186	0.1517
GO0009408 RESPONSE TO HEAT	23	0	0.3348	0.6188	**-2.4766**	0.0120	0.1155
GO0006929 SUBSTRATE BOUND CELL MIGRATION	6	0	0.0404	0.1821	**-2.5390**	0.0083	0.0911
GO0007417 CENTRAL NERVOUS SYSTEM DEVELOPMENT	84	0	0.8493	0.9271	**-2.5593**	0.0335	0.2142
GO0006950 RESPONSE TO STRESS	111	0	0.0590	0.2316	**-2.6652**	0.0006	0.0117
GO0007517 MUSCLE DEVELOPMENT	77	0	0.6424	0.8233	**-2.6960**	0.0069	0.0813
GO0048016 INOSITOL PHOSPHATE MEDIATED SIGNALING	6	0	0.0538	0.2166	**-2.6962**	0.0391	0.2344
GO0008217 BLOOD PRESSURE REGULATION	25	0	0.5149	0.7472	**-2.9581**	0.0009	0.0173
GO0001525 ANGIOGENESIS	111	0	0.5825	0.7879	**-3.0009**	0.0001	0.0020
GO0017156 CALCIUM ION DEPENDENT EXOCYTOSIS	11	0	0.0035	0.0315	**-3.0205**	0.0278	0.1957
GO0030049 MUSCLE FILAMENT SLIDING	6	0	0.2116	0.4856	**-3.2621**	0.0065	0.0778
GO0006937 REGULATION OF MUSCLE CONTRACTION	19	0	0.6034	0.8012	**-4.4898**	0.0026	0.0391
GO0007186 G PROTEIN COUPLED RECEPTOR PROTEIN SIGNA	1582	0	0.4112	0.6785	**-4.7693**	3.12E-07	1.62E-05

Significance defined as Zscore > 1.5 in either direction, p value ≤ 0.05, and false discovery rate (fdr) with cut-off value of 0.3. **Bold font**, significantly up- or down-modulated biological processes. CON, sedentary controls. Related to Figure 4d, e.

Supplementary Table 6. Validation of the microarray analysis by quantitative real-time PCR.

	ΔΔCT relative expression vs 18s					
Primer set	CON-A	HIIT-A	MICT-A	CON-O	HIIT-O	MICT-O
Adam8	1.491	0.533	6.093	37.515	1.356	2.338
	1.652	1.792	25.41	10.856	8.172	2.272
	1.137	10.905	4.298	0.625	6.279	1.128
	0.357	0.285	0.241	6.161	5.201	0.881
Fbxo32	1.334	0.803	0.914	0.306	5.631	8.828
	0.568	0.716	0.84	0.493	4.82	4.094
	1.07	0.603	1.286	0.474	0.499	1.991
	1.232	0.715	1.255	0.496	0.494	1.132
Trim63	0.43	33.12	----	----	85.255	54.015
	3.453	2.407	----	1.658	2.865	15.123
	0.349	0.837	1.912	7.765	0.487	6.397
	1.93	19.573	7.147	41.185	0.563	1.659
Pdk4	0.816	1.48	0.923	0.218	12.777	22.567
	1.397	2.779	0.175	0.244	4.81	6.793
	0.924	0.779	1.323	0.737	0.351	2.757
	0.949	1.938	1.318	0.58	0.251	0.798
Ddit4	0.798	0.882	7.367	4.703	6.299	34.125
	0.488	0.846	20.024	7.096	5.62	22.932
	1.813	2.887	10.41	1.018	2.698	1.631
	1.417	1.096	0.795	1.87	9.76	1.567
Myog1	0.713	0.479	3.334	16.835	0.734	0.901
	1.8	1.174	3.647	14.425	7.178	0.668
	0.894	3.191	0.351	1.051	1.739	1.315
	0.872	0.459	0.288	2.536	0.379	0.913
Ccl2	0.379	0.511	6.361	6.124	0.41	0.202
	3.763	2.614	2.95	5.275	2.567	0.369
	1.241	6.949	4.132	0.313	3.375	0.55
	0.565	0.094	0.131	0.726	3.871	0.341
Col1a1	0.601	0.568	2.959	1.641	0.41	0.067
	1.723	1.425	1.969	1.791	0.679	0.234
	1.268	3.13	1.249	0.715	1.871	0.23
	0.761	0.467	0.34	2.228	1.972	0.424

n = 4 mice per experimental group. Abbreviations: CON, sedentary controls; HIIT, high-intensity interval training; MICT, moderate-intensity continuous training. -A, adult; -O, old. Related to Figure 4h and Supplementary Figure 6b.

Supplementary Table 7. Detection of inflammatory cytokines in skeletal muscle extracts by multiplex analysis.

		CONT (n=5)		HIIT (n=5)		MICT (n=5)	
	Cohort	Mean	SEM	Mean	SEM	Mean	SEM
IFNγ	Adult	B.D.		B.D.		B.D.	
	Old	B.D.		B.D.		B.D.	
IL-1β	Adult	0.116	0.014	0.138	0.032	0.255	0.139
	Old	0.190	0.044	0.173	0.024	0.326	0.133
IL-4	Adult	0.073	0.014	0.068	0.009	0.036	0.008
	Old	0.094	0.023	0.097	0.027	0.059	0.005
IL-5	Adult	0.123	0.010	0.136	0.007	0.114	0.006
	Old	0.152	0.017	0.139	0.017	0.153	0.019
IL-6	Adult	6.762	0.919	6.913	0.269	9.785	0.898
	Old	5.526	0.159	7.351	0.692	6.748	1.254
KC/GRO	Adult	0.702	0.030	0.724	0.129	0.556	0.050
	Old	1.069	0.074	1.294	0.314	1.336	0.068
IL-10	Adult	1.485	0.137	1.706	0.115	1.186	0.092
	Old	1.237	0.071	2.172	0.361	1.282	0.095
IL-12/p70	Adult	11.062	1.934	10.576	1.264	10.667	0.661
	Old	13.366	1.168	13.902	1.312	10.261	1.357
TNFα	Adult	0.241	0.048	0.283	0.073	0.378	0.193
	Old	0.495	0.111	0.354	0.067	0.499	0.086

Abbreviations: CONT, sedentary controls; HIIT, high-intensity interval training; MICT, moderate-intensity continuous training; B.D. below detection. All values are expressed as pg/ml per well. Related to Supplementary Figure 6c.

Supplementary Table 8. Effect of age, exercise and 'age x exercise' interaction on skeletal muscle cytokine levels obtained by multiplex assay.

Analyte		F (DFn, DFd)	P value	Analyte	F (DFn, DFd)	P value
IL-1β	Interaction	(2,23) = 0.0398	0.9611	KC/GRO	(2,24) = 1.797	0.1874
	Age	(1,23) = 0.9026	0.3520		(1,24) = 11.82	**0.0021**
	Exercise	(2,23) = 1.999	0.1584		(2,24) = 0.8090	0.4571
IL4	Interaction	(2,24) = 0.0387	0.9621	IL10	(2,24) = 2.079	0.1470
	Age	(1,24) = 3.180	0.0872		(1,24) = 0.5344	0.4718
	Exercise	(2,24) = 3.154	0.0608		(2,24) = 9.185	**0.0011**
IL5	Interaction	(2,24) = 0.8799	0.4278	IL12/p70	(2,24) = 0.3074	0.7382
	Age	(1,24) = 4.499	**0.0444**		(1,24) = 0.3967	0.5348
	Exercise	(2,24) = 0.0518	0.9497		(2,24) = 0.5409	0.5892
IL6	Interaction	(2,24) = 2.386	0.1135	TNFα	(2,24) = 0.3884	0.6823
	Age	(1,24) = 3.872	0.0608		(1,24) = 2.906	0.1012
	Exercise	(2,24) = 3.562	**0.0442**		(2,24) = 0.6351	0.5385

Statistical analysis was performed by two-way ANOVA coupled with Sidaks's post-hoc text. Related to Supplementary Figure 6c and Supplementary Table 7. Bold font, significant difference at $p < 0.05$.

Supplementary Table 9. Lysis cocktail for protein homogenization.

Description	Vendor	Cat #	Dilution
TSA (Trichostatin A)	Sigma-Aldrich	T1952	1:500
PMSF (Phenylmethanesulfonyl fluoride)	Sigma-Aldrich	P7626	1:1,000
Protease Inhibitor Cocktail	Sigma-Aldrich	P8340-5ML	1:100
Phosphatase Inhibitor Cocktail 2	Sigma-Aldrich	P5726-5ML	1:100
Phosphatase Inhibitor Cocktail 3	Sigma-Aldrich	P0044-5ML	1:100
1M Nicotinamide	Sigma-Aldrich	47865-U	1:100
5M Sodium butyrate	Sigma-Aldrich	303410-100G	1:10,000

Supplementary Table 10. Antibodies used for immunoblotting.

Name	Host	Vendor	Serial #	Milk/BSA	Dilution
LC3B	Rabbit	Cell Signaling Technology (CST)	2775S	Milk 5%	1:1000
PGC-1α	Rabbit	Abcam	ab54481	Milk 5%	1:1000
HKII	Rabbit	CST	2867S	Milk 5%	1:1000
FIS1	Rabbit	Santa Cruz Biotechnology (SCB)	sc-98900	Milk 5%	1:1000
LKB1	Rabbit	CST	3047S	Milk 5%	1:1000
IRS1	Rabbit	CST	2382S	Milk 5%	1:1000
PKM1	Rabbit	Abcam	ab38237	Milk 5%	1:1000
OXPHOS Rodent Cocktail	Mouse	Abcam	ab110413	Milk 5%	1:1000
CPT Ib	Rabbit	Lifespan Biosciences	LS-C12435	Milk 5%	1:1000
VDAC1/Porin	Rabbit	Abcam	ab15895	Milk 5%	1:1000
HADHSC	Goat	SCB	sc-74650	Milk 5%	1:1000
SIRT3	Rabbit	CST	5490	Milk 5%	1:1000
SIRT1	Mouse	Sigma-Aldrich	S5196	Milk 5%	1:1000
Fibronectin 1	Rabbit	Millipore-Sigma	F3648	Milk 5%	1:1000
Collagen III	Rabbit	Abcam	Ab7778	Milk 5%	1:1000
α-SMA	Mouse	Millipore-Sigma	A5228	Milk 5%	1:2000
Vinculin	Mouse	Millipore-Sigma	V9131	Milk 5%	1:2000
Bovine anti-rabbit IgG-HRP		SCB	sc-2370	Milk 5%	1:5000
Bovine anti-goat IgG HRP		SCB	sc-2350	Milk 5%	1:5000
Bovine anti-mouse IgG HRP		SCB	sc-2371	Milk 5%	1:5000

Supplementary Table 11. List of murine oligonucleotide primers used for validation of microarray analysis by quantitative real-time PCR.

Accession number	Target mRNA	Amplicon Size (nt)	Primer orientation	Sequence (5' -> 3')
NM_007403	Adam8	100	Forward<br>Reverse	AGTTCCTGTTTATGCCCCAAAG<br>AAAGGTTGGCTTGACCTGCT
NM_026346	Fbxo32	244	Forward<br>Reverse	CAGCTTCGTGAGCGACCTC<br>GGCAGTCGAGAAGTCCAGTC
NM_001039048	Trim63	234	Forward<br>Reverse	CCAGGCTGCGAATCCCTAC<br>ATTTTCTCGTCTTCGTGTTCCTT
NM_013743	Pdk4	185	Forward<br>Reverse	AGGGAGGTCGAGCTGTTCTC<br>GGAGTGTTCACTAAGCGGTCA
NM_029083	Ddit4	107	Forward<br>Reverse	GTGCTGCGTCTGGACTCTC<br>CCGGTACTTAGCGTCAGGG
NM_178440	Myog1	128	Forward<br>Reverse	GGCCCTGAGTATGGGAAACC<br>GATACGAGCACCTCACCAATG
NM_011333	Ccl2	120	Forward<br>Reverse	TAAAAACCTGGATCGGAACCAAA<br>GCATTAGCTTCAGATTTACGGGT
NM_007742	Col1a1	103	Forward<br>Reverse	GCTCCTCTTAGGGGCCACT<br>CCACGTCTCACCATTGGGG
NR_003278	Rn18s	99	Forward<br>Reverse	TTCCGATAACGAACGAGACTCT<br>TGGCTGAACGCCACTTGTC
NM_007393.5	β-actin	151	Forward<br>Reverse	TGAGAGGGAAATCGTGCGTGAC<br>CCGCTCGTTGCCAATAGTGATG

Related to Figure 4h, Supplementary Figure 6b and Supplementary Table 6.
